# RNA Interference of Three Genes of the Unfolded Protein Response: Activating Factor of Transcription 4, Eukaryotic Translation Initiation Factor 2-alpha Kinase, and Inositol-Requiring Enzyme 1 in Acyrthosiphon pisum

**Published:** 2022-08-29

**Authors:** Jared Ridder, James Balthazor

**Affiliations:** Department of Chemistry, Fort Hays State University, USA

**Keywords:** Pea aphid, dsRNA, UPR proteins, Transcripts, Genes, Transcriptomics, Proteomics, Silencing

## Abstract

The Activating Transcription Factor 4 (ATF4) is a transcription factor that can act as both an activator and repressor and is a critical component of the Unfolded Protein Response (UPR) and Amino Acid Response (AAR) pathways. Inositol-requiring enzyme 1 (IRE1) is an Endoplasmic Reticulum (ER) membrane bound kinase/endoribonuclease that functions as a sensor of unfolded protein and is the most conserved component of the UPR in eukaryotes. Eukaryotic Initiation Factor-2-alpha Kinase (PERK) is an ER membrane bound kinase that phosphorylates eukaryotic initiation factor upon activation of the UPR causing downregulation of protein synthesis. It was hypothesized that introduction of double stranded RNA (dsRNA) complementary to each of the target genes to aphid diet may correlate with a change in expression of each gene. The objective of this study was to determine the possible relationship between fed dsRNA concentration and aphid survival. Increase in concentration of ATF4 dsRNA in artificial diet was correlated to a decrease in survival of fed aphids. Greater concentrations of fed dsRNA were associated with less expression of ATF4 mRNA in whole aphids. Increase in concentration of IRE1 and PERK dsRNAs in artificial diet were not correlated to a decrease in survival of fed aphids, although increase in concentrations of the respective dsRNAs were associated with less expression of the target gene mRNAs. These results suggest that target mRNA expression appears to be influenced by concentration of fed dsRNA. The results of this study also indicate that decrease in ATF4 expression is associated with decreased insect survival while decrease in IRE1 and PERK expression is not.

## INTRODUCTION

Regulation of genes of the Unfolded Protein Response (UPR) is a complex chemical process that is dependent on multiple feedback loops, coordinated enzyme action, and harmonious organelle interaction to accurately transfer information stored in genetic sequences and produce functional protein [1]. Transcription of DNA is regulated by transcription factors which enhance or inhibit the action of RNA polymerase II. Transcribed pre-mRNAs are transported to the spliceosome, where they are spliced into mature mRNAs [2]. The mRNAs are translated at the ribosome to produce new peptide chains. If the new peptide contains a signaling sequence, a stretch of hydrophobic amino acids approximately 5–16 residues in length, at its N-terminus, the ribosome/peptide/mRNA complex is directed to merge with the membrane of the endoplasmic reticulum (ER) [3]. Proteins destined for secretion are modified post-translation within the lumen of the ER. Proteins modified within the ER are transformed by means of: Formation of disulfide bonds, folding mediated by chaperones, site-specific glycosylation, site-specific proteolysis, and assembly of monomers into multimeric proteins [4]. ER-associated protein machinery is finite, and if upstream pathways are upregulated, the ER can become overwhelmed, resulting in negative consequences for the affected cell. The ER adapts to flux in protein production demand by means of the UPR [5]. The UPR is a suite of genes that transduce information to the nucleus about the internal status of protein production and aggregation within the lumen of the ER. It is an adaptive mechanism that responds to unfolded proteins within the ER lumen [6]. The UPR adaptively regulates the expression of genes to maintain proteostasis within the ER or induces apoptosis if ER stress is unresolved [7]. The UPR responds to stress by upregulating chaperone proteins, inducing ER autophagy, degrading mRNA proximal to the ER, attenuating the rate of mRNA transcription and translation, and inducing apoptosis if the response is overwhelmed [8]. There are three main ER transmembrane signaling proteins that respond to the accumulation of misfolded proteins within the ER: Cyclic AMP-Dependent Transcription Factor 6 (ATF6), Inositol-Requiring Enzyme 1 (IRE1), and Eukaryotic Initiation Factor-2-Alpha Kinase (PERK) [9]. Under normal conditions, these signal transducers are held in an inactive conformation by binding to Binding Immunoglobulin Protein (BiP), also known as GRP78. However, when misfolded protein within the ER lumen accumulates, BiP is titrated away from the signal transducers, and the UPR is activated [10]. Upregulation of UPR components has been associated with various neurodegenerative diseases, cancer, diabetes mellitus, and viral infections [11,12]. This study investigates 3 genes of the UPR: Activating Transcription Factor 4 (ATF4), IRE1, and PERK.

ATF4 is an evolutionarily conserved transcription factor that acts as both an activator and repressor of transcription [11]. In humans, the ATF4 gene is located on chromosome 22. ATF4 belongs to the Cyclic AMP Response Element Binding protein (CREB) family of Basic Leucine Zipper (bZIP) transcription factors, and the activity of its gene is associated with various functions, including expression of genes involved in oxidative stress response, amino acid synthesis, and cell differentiation [13]. The expression of ATF4 is upregulated in response to oxidative stress, amino acid deficit, and prevalence of unfolded proteins within the lumen of the ER [14]. Upregulation of ATF4 promotes transcription of chaperone proteins, macro autophagy of affected ER segments, amino acid metabolism, and in terminally damaged cells, it induces apoptosis [11]. In humans, upregulation of ATF4 protein was observed in tach paced, oxygen deficient cardiomyocytes, resulting in inflammation and cell death. However, induced ATF4 overexpression in resting state cardiomyocytes caused upregulation of genes responsible for amino acid biosynthesis, primarily asparagine synthetase [15,16]. Based on observations in humans, it is possible that changes in expression of ATF4 might contribute to cell viability and subsequent changes in lifespan of pea aphids. However, the link between ATF4 expression and aphid survival has not been explored.

IRE1 is the most evolutionarily conserved component of the UPR in eukaryotes. IRE1 is an ER transmembrane kinase/endoribonuclease that functions as a sensor of unfolded protein within the ER lumen [5]. In humans, the IRE1 gene is located on chromosome 17. In normally functioning cells, BiP is bound to the luminal, N-terminus kinase domain of IRE1, preventing function. In the event of protein misfolding within ER, BiP disassociates from IRE1 to act as a chaperone. IRE1 undergoes dimerization and auto-phosphorylation, resulting in the exposure of the cytosolic endoribonuclease domain [17]. The main substrate of IRE1 is the X-box binding protein 1 (Xbp1) pre-mRNA [11]. Mature Xbp1 translocates to the nucleus and induces transcription of genes encoding ER-Associated Degradation (ERAD), and modulates phospholipid synthesis necessary for ER expansion while under stress. In instances of unresolved ER stress, IRE1 monomers form large clusters which participate in Regulated IRE1-Dependent Decay (RIDD). These RIDD clusters cleave cytosolic mRNA proximal to the ER, ultimately reducing the amount of protein that can enter the ER [18]. It is possible that changes in expression of IRE1 may contribute to changes in lifespan of pea aphids.

PERK is an ER transmembrane kinase that functions as a sensor of unfolded protein within the ER lumen [5]. In humans, the PERK gene is located on chromosome 2. In normally functioning cells, BiP is bound to the luminal domain of PERK preventing function. Unfolded protein within the ER titrates BiP away from PERK, resulting in PERK monomer dimerization and auto-phosphorylation and exposure of the cytosolic kinase domain [19]. PERK phosphorylates the alpha subunit of eukaryotic initiation factor 2 (eIF2α). The phosphorylation of eIF2α causes ribosome turnover rates to diminish through the binding of guanine nucleotide exchange factor, preventing the 40’s ribosomal subunit from properly assembling. This interaction decreases the rate of protein production, and allows the ER to clear accumulated misfolded protein, increasing the chance of cell survival [5]. Phosphorylated eIF2α also serves as a positive transcription factor of ATF4, leading to an increase in ATF4 mRNA concentration and all subsequent responses described [20]. It is possible that changes in expression of PERK may contribute to changes in lifespan of pea aphids.

RNA interference (RNAi) is a method of gene silencing that is achieved In vivo upon production or introduction of double stranded RNA (dsRNA) in targeted cells [21] dsRNA in the cytoplasm is cleaved by the enzyme DICER into double stranded segments 20–22 nucleotides in length [22]. These small dsRNAs are bound to an argonaut protein, a helicase removes one of the RNA strands, and the RNA/argonaut complex is then active [23]. If the RNA/argonaut complex encounters a mature mRNA molecule with a complementary sequence, the complex associates with the mRNA and recruits other proteins to form the RNA-Induced Silencing Complex (RISC). The RISC is an endonuclease that hydrolyzes the mRNA, preventing translation and silencing the target gene [24]. Previous studies have demonstrated successful methods of feeding dsRNAs to insects to cause gene knockdown. Successful RNAi mediated gene knockdown by fed dsRNA has been observed in pea aphids [25,26], (red flour beetles [26,27] mosquitos [28], and fruit flies) [29]. In aphids, previous RNAi mediated knockdown of gene products has been associated with decreased survival rates. Few studies have been conducted focusing on RNAi of the UPR in pea aphids [25]. However, the exact mechanisms associated with introduction of dsRNA complementary to mRNA of UPR genes is unknown in pea aphids.

Based on observations in other RNAi knockdown studies in pea aphids, it is possible that knockdown in expression of ATF4, IRE1, and PERK may contribute to changes in survival time of pea aphids. However, the link between anti-UPR gene dsRNA and the change in expression of the target UPR gene has not been previously explored.

Therefore, the objectives of this study are to examine the possible relationship between introduction of anti-gene dsRNAs complementary to ATF4, IRE1, and PERK mRNAs to the diet of pea aphids and the change in survival in pea aphids, as well as the change in expression of ATF4, IRE1, and PERK mRNA in pea aphids.

## MATERIALS AND METHODS

### Insect Care and Maintenance

The aphid colony was obtained from Kansas State University, Department of Biochemistry and Molecular Biophysics, Manhattan, KS, USA. Aphids were reared in commercially available BugDorms (BioQuip Products, Rancho Dominguez, CA, USA). 12 inch by 16 inch plastic trays were placed in the BugDorms to hold self-watering plant pots. Aphids were maintained on budding Vicia faba plants (Mountain Valley Seed Co., Salt Lake City, UT, USA) which were replaced twice weekly. Vicia faba plants were grown in commercially available soil (Gardener’s Supply Company, Burlington, VT, USA), contained in commercially available plastic planters (Gardener’s Supply Company, Burlington, VT, USA). Aphids and plants were maintained in a 12:12 light:dark photocycle under full-spectrum growth light.

### Identification of Pea Aphid ATF4 Gene

The ATF4 transcript was identified by searching the pea aphid genome database available in GenBank. Human UPR transcripts were used as a query using BLASTn to find orthologous transcripts in the pea aphid. One ATF4 transcript variant was identified for RNAi study (GenBank Accession Number: XM_008189240.2) from a comparison of known human ATF4 (NP_001666.2). To identify the locus of ATF4 within pea aphid chromosomes, the transcript was compared against pea aphid genome sequences available in GenBank with the RefSeq blast function. The predicted amino acid sequence of the transcript was compared to sequences in GenBank with the blastp function. The pea aphid ATF4 transcript was compared against multiple model organism transcripts: One human (NP_001666.2), one mouse (NP_001274109.1), one pig (XP_020946546.1), one anole (XP_003221029.1), one zebrafish (XP_005172112.1), one aphid (XP_015363901.), one fruit fly (NP_001260672.1), and one roundworm (NP_510456.1) transcript variants. Multiple alignments of ATF4 proteins, as well as a phylogenetic tree that demonstrated the evolutionary relationship among aphid ATF4 and ATF4 among other model organisms, were generated with Geneious Software. A set of double stranded RNA (dsRNA) synthesis primers, containing the T7 promoter sequence (forward: TAATACGACTCACTATAGGGACGGCGAGTGCCAATATG, reverse: TAATACGACTCACTATAGGGAATCTTCTTTCTCGTCAACAACC) and a set of quantitative real time polymerase chain reaction (qRT-PCR) primers (forward: CACTTATGACCCCGTAAGCC, reverse: GGAAGCCATATTGGCACTCG) were designed based on the pea aphid ATF4 X1 transcript variant sequence (Integrated DNA Technologies, INC., 1710 Commercial Park, Coralville, IA, USA). The primer sets were designed to cover an exon-exon junction.

### Identification of Pea Aphid IRE1 Gene

The IRE1 transcript was identified by the method described in Section 2.2. One IRE1 transcript variant was identified for RNAi study (GenBank Accession Number: XP_001943673). The pea aphid IRE1 transcript was compared against multiple model organism transcripts: One human (NP_001424), one mouse (NP_076402), one pig (XP_005668752), one anole (XP_003229691), one zebrafish (NP_001919350), one aphid (XP_015365603), one fruit fly (NP_001097839), and one roundworm (NP_001254135) transcript variants. Multiple alignments of IRE1 proteins, as well as a phylogenetic tree that demonstrated the evolutionary relationship among aphid IRE1 and IRE1 among other model organisms, were generated as described in Section 2.2. A set of dsRNA synthesis (forward: TAATACGACTCACTATAGGGTGCGCTGAAATTCTGTTTACTGT, reverse: TAATACGACTCACTATAGGGGGCCAATGCCATTTTGTCGT) and qRT-PCR primers (forward: CATTATTACAAAAAGGTGTTCAGCG, reverse: CCAGACGAGATGGTGGTAGC) were designed as described in Section 2.2.

### Identification of Pea Aphid PERK Gene

The PERK transcript was identified by the method described in chapter 2.2. One PERK transcript variant was identified for RNAi study (GenBank Accession Number: XM_001947026). The pea aphid PERK transcript was compared against multiple model organism transcripts: One human (NP_055228), one mouse (NP_001300847), one pig (XP_003124973), one anole (XP_003222450), one zebrafish (NP_001107942), one aphid (XP_015364823), one fruit fly (NP_001263141), and one roundworm (NP_509912) transcript variants. Multiple alignments of PERK proteins, as well as a phylogenetic tree that demonstrated the evolutionary relationship among aphid PERK and PERK among other model organisms, were generated as described in Section 2.2. A set dsRNA synthesis (forward: TAATACGACTCACTATAGGGCCAATACCATAGCGAAACAATA, reverse: TAATACGACTCACTATAGGGATAACAAAGCGATACCATAACC) and qRT-PCR primers (forward: TGTCCGAGCATCAGACACAC, reverse: TGGGAGACTCCGATTTGTGAG) were designed as described.

### Total Cellular RNA Isolation and Synthesis of Complementary DNA (Cdna)

Benchtops were sterilized with RNase Away Reagent (Thermo Fisher Scientific, Waltham, MA, USA) according to manufacturer’s instructions. To extract total cellular RNA, 10 adult pea aphids were transferred into a 1.5 mL RNase-free micro centrifuge tube (Thermo Fisher Scientific) and homogenized in 1.0 mL of TRIzol Reagent (Thermo Fisher Scientific, lot#177301). The sample was spun in a refrigerated centrifuge at 12,000 × g at 4°C for 10 minutes. The sample was decanted into a clean 1.5 mL micro centrifuge tube and 200 μL of chloroform (Thermo Fisher Scientific, lot#050309) were added and vortexed. The sample was spun in a refrigerated centrifuge at 12,000 × g at 4°C for 10 minutes. The upper aqueous layer was removed by pipette and transferred into a clean micro centrifuge tube containing 500 μL cold isopropanol (Thermo Fisher Scientific, lot#127567) and incubated on ice for 5 minutes to facilitate RNA precipitation. The sample was spun in a refrigerated centrifuge at 15,000 × g and 4°C for 10 minutes. The liquid was decanted, and the pellet was washed with 100 μL of cold absolute ethanol (Decon Laboratories INC., ref#2716, King of Prussia, PA, USA). The ethanol was decanted, and the pellet was incubated at room temperature until the residual ethanol evaporated. Fifty μL of RNase-free water (Thermo Fisher Scientific, ref#10977–015) were added to the pellet, and the sample was incubated at 37°C for one minute to facilitate RNA solvation. Isolated RNA was treated with commercially available DNase I (TURBO DNA-freeTM; Thermo Fisher Scientific cat#1725085) according to manufacturer’s instructions to remove genomic DNA contamination. The quantity of DNase I treated RNA was measured by measuring UV absorbance at 260 nm using a Nano drop One spectrophotometer (Thermo Fisher Scientific). Quality of RNA was estimated by calculating the ratio of UV absorbance at 260 nm and 280 nm. Only samples with an A260/A280 ratio greater than 1.90 were considered for cDNA synthesis. All RNA was stored at −40°C until cDNA synthesis.

An iScript DNA synthesis kit (Bio-Rad Corporation, cat#1725085, Hercules, CA, USA) was used to synthesize cDNA from 1.0 μg of DNase I treated total cellular RNA according to manufacturer’s instructions. Quantity and quality of cDNA were measured by UV absorbance as described above. The cDNA was stored at −40°C until dsRNA synthesis.

### Synthesis of Anti-ATF4, Anti-IRE1, and Anti-PERK dsRNAs

A T7 RNA polymerase kit (Bio-Rad Corporation, lot#00614019) was used to synthesize dsRNA from 1.0 μg of cDNA and 1 μL each of forward and reverse primers (10 pmol/μL) for each anti-gene dsRNA according to manufacturer’s instructions. Quantity and quality of dsRNA were measured by UV absorbance as described in Section

### The dsRNA was Stored At −40°C until Feeding Studies

Preparation of dsRNA-branch amphiphilic peptide capsule (BAPC) nanoparticle containing diet: By using the 2-ΔCT method described previously to Akey and Beck, 1971 [30]. 1 μg of anti-ATF4 dsRNA was dissolved in 10 μL of RNase-free water. The dsRNA solution was added drop wise into a 10 μL solution containing 200 μM BAPCs according to [26] and incubated at room temperature for 10 minutes before adding enough CaCl2 to yield a concentration of 1.0 mM CaCl2. After 10 minutes incubation, the solution was diluted with Akey-Beck diet to 100 μL. For insects treated with lesser amounts of anti-gene dsRNA, BAPC/nucleotide complexes prepared above were diluted 10x and 100x with Akey-Beck diet. This procedure was repeated with anti-IRE1 and anti-PERK dsRNAs.

### Effects of Variable dsRNA Concentration on Insect Lifespan

For negative control samples, 50 adult pea aphids were placed on each of three petri dishes. A layer of stretched parafilm (Thermo Fisher Scientific) was placed over each dish. 100 μL of Akey-Beck diet were placed on top of the parafilm, and another layer of parafilm was stretched over the diet to form a pocket. Aphids fed on the diet by penetrating the bottom layer of parafilm with a piercing action. Aphids were fed on Akey-Beck diet for 48 hours; the diet was removed, healthy Vicia faba leaves were inserted into the petri dishes, and they were resealed with parafilm.

Diets containing various concentrations of anti-gene dsRNAs prepared as described in Section 2.7 were fed to aphids as described above. Aphids fed on dsRNA-containing diet for 48 hours and were transferred to plant leaves as previously mentioned. Three replicates were performed for each feeding study. Survival of each experimental group was monitored every three hours to record and remove dead adult aphids and nymphs.

### Treatment Aphid RNA Extraction and cDNA Synthesis

To prepare for qPCR analysis, another feeding study was prepared as described in Section 2.8. Two aphids from each treatment group were removed from feeding every twelve hours until 48 hours had elapsed. Total cellular RNA was isolated from each group and used to prepare cDNA as described in Section 2.5 cDNA from dsRNA-fed aphids was stored at −40°C until used in qPCR assays.

### Real-Time qRT-PCR

Expression of ATF4, PERK, and IRE1 was measured by qRT-PCR using SYBR green technology. The primers used for gene assays were designed based on sequences identified in Sections 2.2, 2.3 and 2.4 (Table 1). The ribosomal protein L27 (RPL27) gene (forward: TCGTTACCCTCGGAAAGTC, reverse: GTTGGCATAAGGTGGTTGT) was used as an internal positive control for examination of target gene knockdown. The reaction solutions for qRT-PCR consisted of 10 μL SSoAdvanced SYBR Green Supermix (Bio-rad Corporation, lot#1725085), 1 μL of 10 μM gene specific forward primer, 1 μL of 10 μM gene specific reverse primer, 5 μL of 10 ng/μL treatment specific cDNA, and 5 μL of RNase-free water. In positive control wells, 5 μL of RNase-free water were added in place of cDNA. The final volume of the reaction solution was 20 μL per well. Bio-Rad CFX96 real time detection system (Bio-Rad Corporation) was used to perform qRT-PCR. Thermo cycle consisted of a hot start (90°C for 3 minutes) followed by 40 cycles of 95°C for 30 seconds, 55°C for 30 seconds, and 72°C for 30 seconds. For examination of target gene expression, the Cycle Threshold (CT) value of the internal control from each sample was subtracted from the CT value of the respective target gene. Expression of total target gene in each sample was calculated by using the 2-ΔCT method described previously [31]. Target gene expression was converted to relative expression by dividing expression of the target gene samples (2-ΔCT) by the lowest expression sample of the target gene. Before statistical analysis, expression of respective target gene transcripts was converted to “change over control” by dividing expression of each target gene sample (2-ΔCT) with average 2-ΔCT values of the respective control groups. The control aphids were fed on artificial diet without dsRNA for 48 hours.

### Statistical Analysis

Statistical analysis of target gene expression during feeding studies was conducted with R (version 3.3.2) using a one-way Analysis of Variance (ANOVA) with treatment (time, concentration) as the independent variable and expression of target gene as the dependent variable. Statistically significant differences between treatments (p<0.05) were confirmed using a Tukey’s test. The data are presented as means ± standard deviation. Statistical analysis of aphid survival during feeding studies was conducted with R (version 3.3.2) using a Log-rank (Mentel-Cox) test with treatment as the independent variable and survival in hours as the dependent variable. When the p<0.05 differences in means were considered statistically significant. When the p<0.10 but greater than 0.05 (p<0.10), differences between means were considered as tendency. Otherwise, differences between means were considered not statistically significant (p>0.10).

## RESULTS

### In Identification of Target Pea Aphid ATF4, IRE1, and PERK Genes

**Figure 1:**
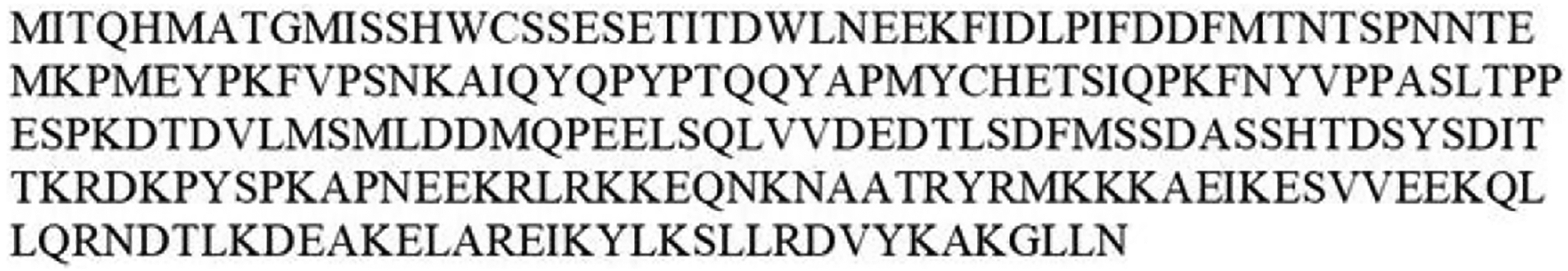
Predicted amino acid sequence of pea aphid ATF4 transcript variant X1 acquired using GenBank.

Screening of GenBank yielded three highly homologous ATF4 sequences found in pea aphids. Figure 1 show the amino acid sequence of ATF4 isoform X1, which was chosen for this study.

**Figure 2:**
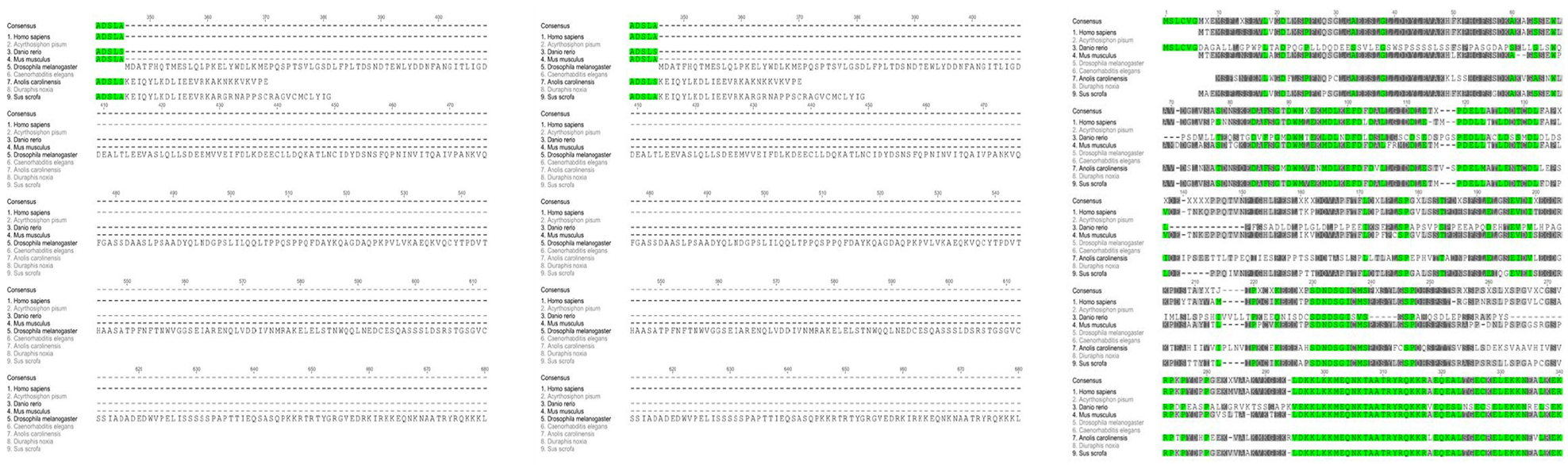

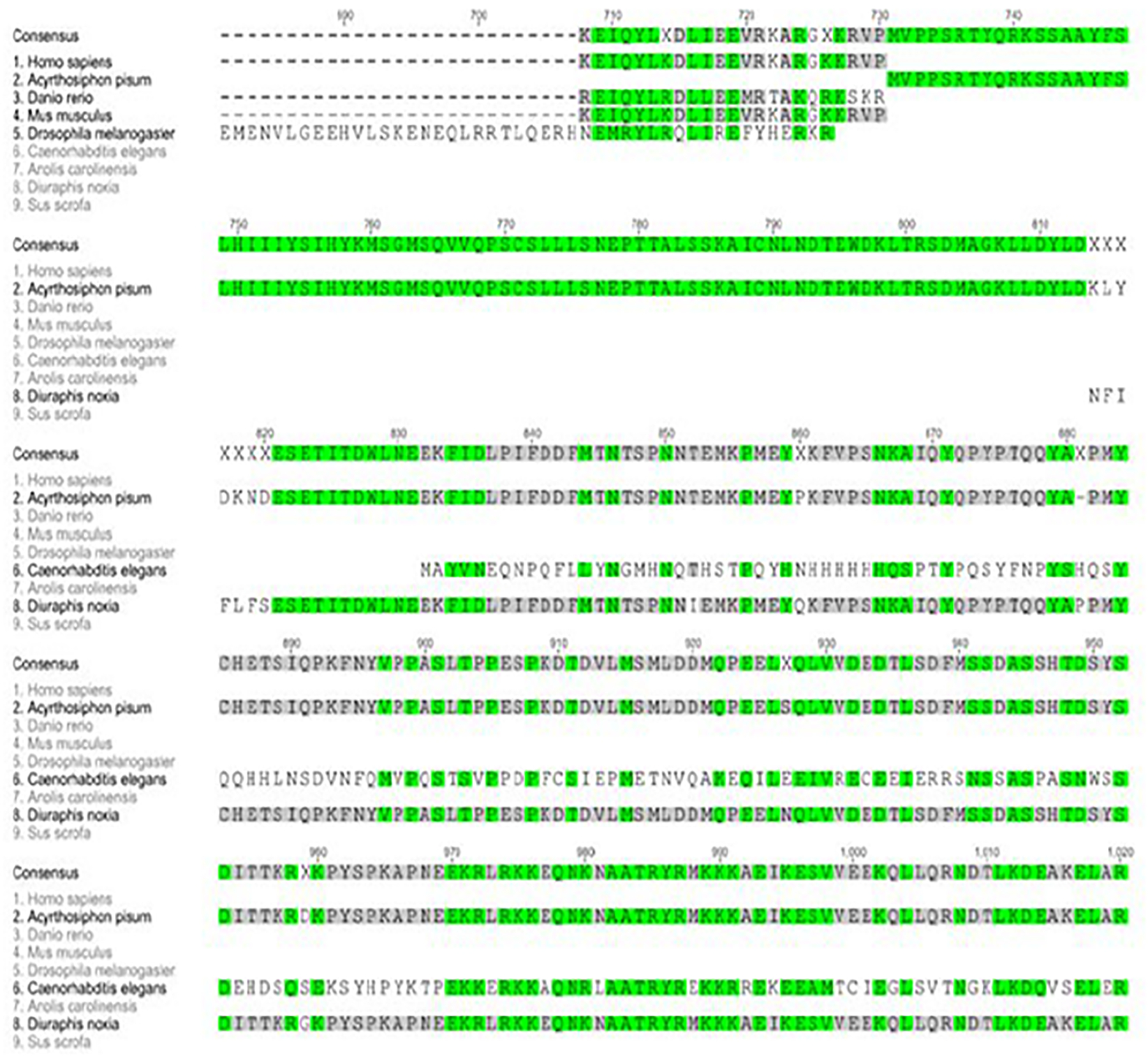
Multiple sequence alignment comparing pea aphid ATF4 amino acid sequence aligned with selected model organisms.

**Figure 3:**
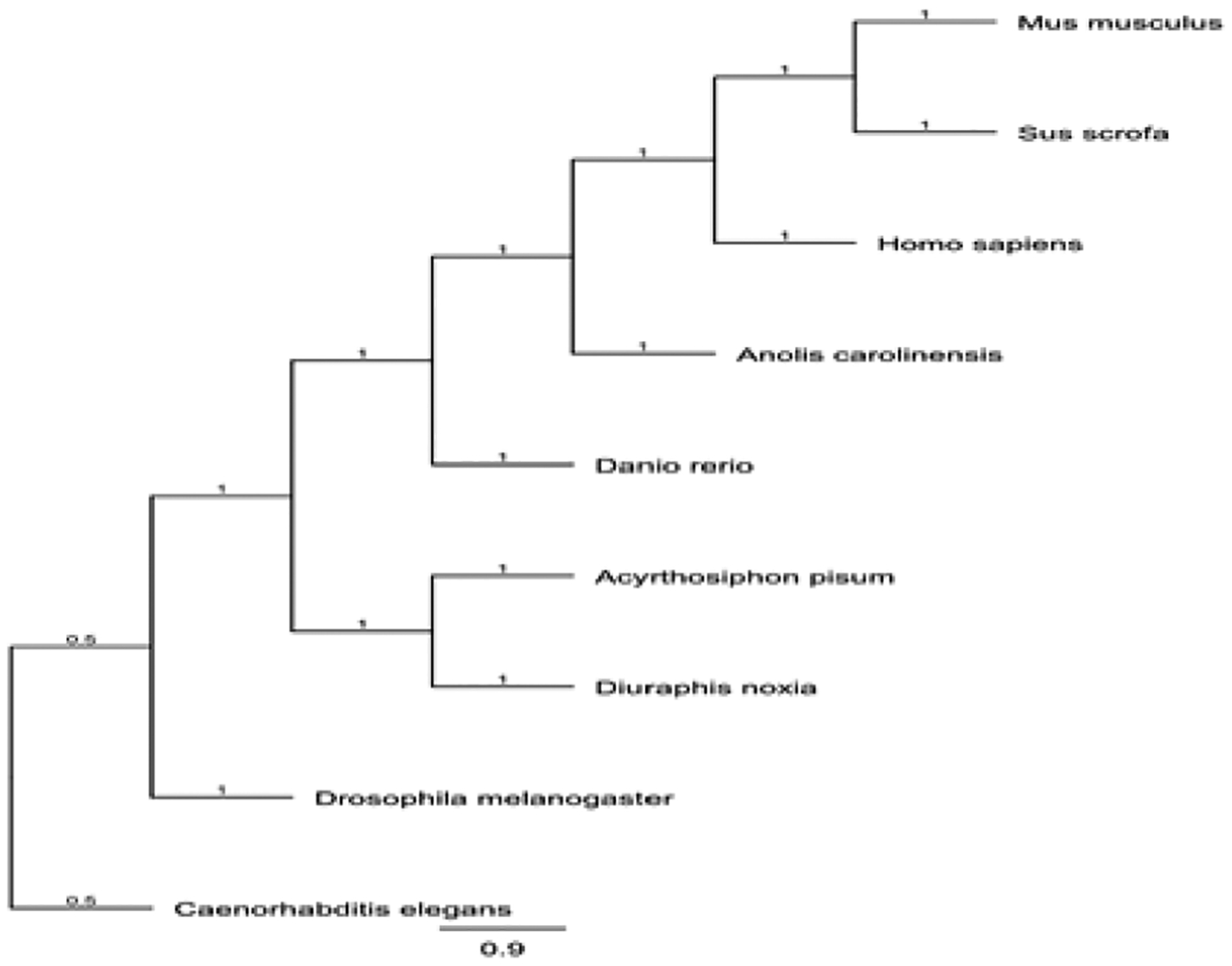
Neighbor-joining phylogenetic tree indicating the evolutionary relationship of pea aphids (*Acyrthosiphon pisum*) ATF4, and selected model organisms.

A multiple sequence alignment and phylogentic tree of ATF4 generated with Genious software are indicated in Figures 2 and 3, respectively. Analysis with the NCBI conserved domain tool demonstrated that the C-terminal contains the Basic Leucine Zipper (bZIP) domain responsible for protein protein interactions and DNA binding. This domain is conserved in ATF4 of other organisms. The comparison of the ATF4 sequence against the pea aphid reference genome sequence database available in GenBank demonstrated that the transcript aligned with an unplaced scaffold within the assembly (GenBank Accession Number: NW_003384491.1).

**Figure 4:**
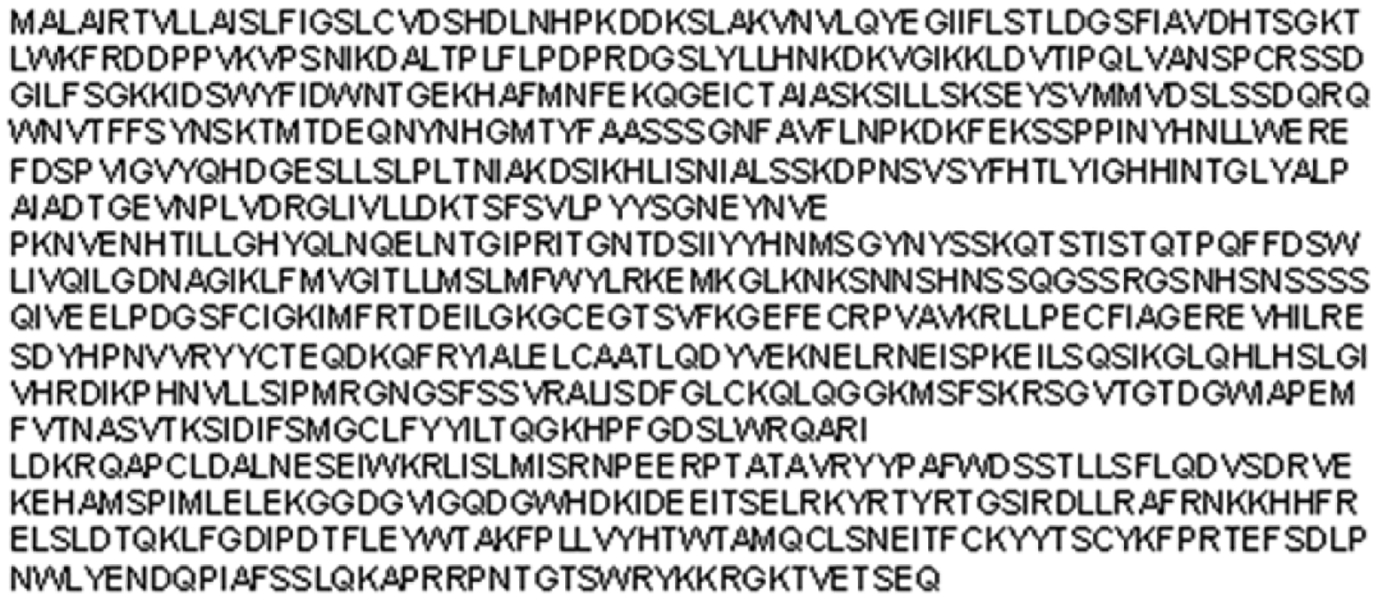
Predicted amino acid sequence of pea aphid IRE1 transcript variant X1 acquired using GenBank.

Screening of GenBank yielded two highly homologous IRE1 sequences found in pea aphids. Figure 4 shows the amino acid sequence of IRE1 isoform X1, which was chosen for this study.

**Figure 5:**
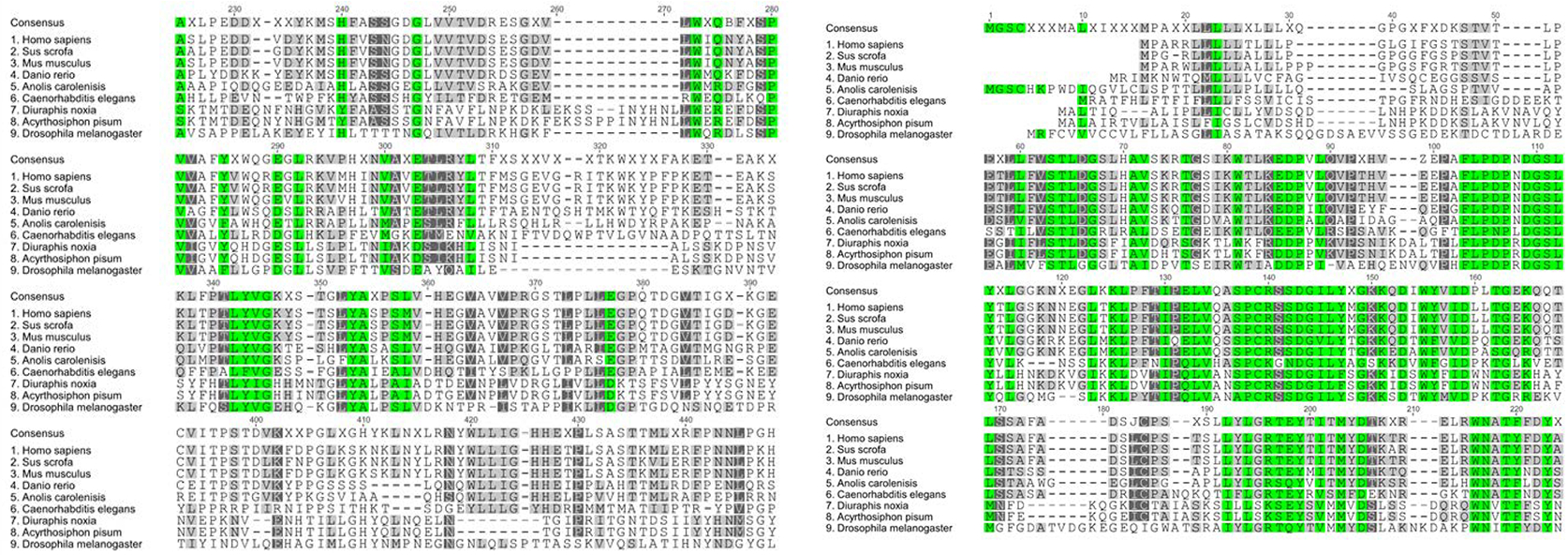

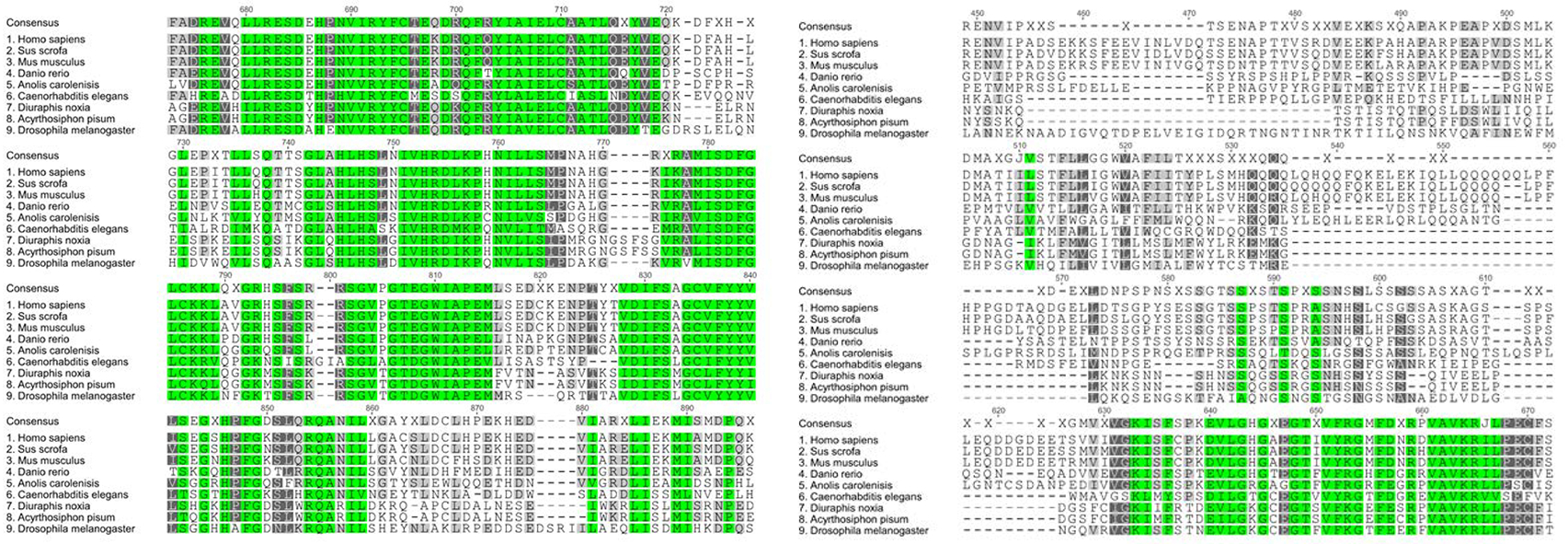

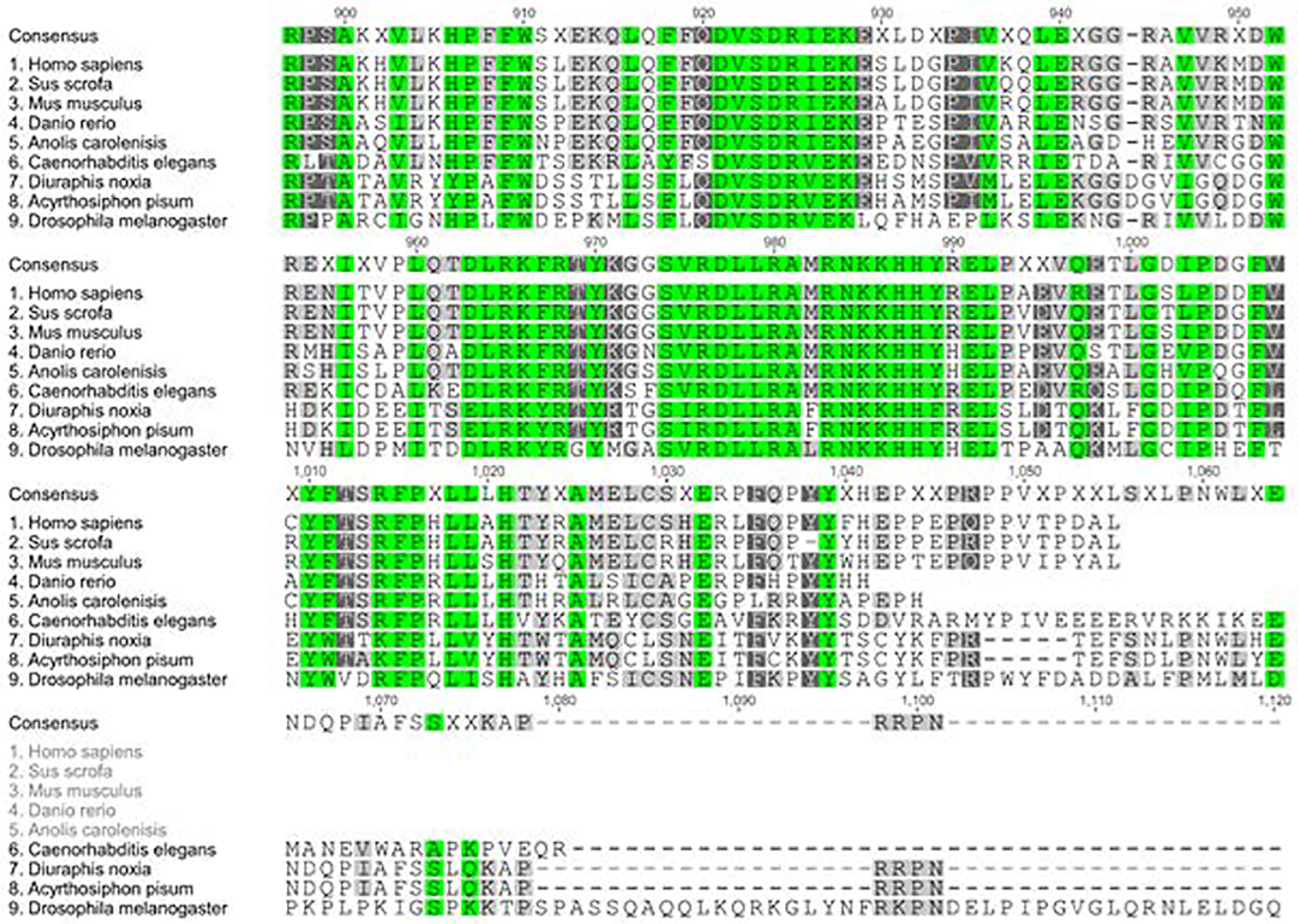
Multiple sequence alignment comparing pea aphid IRE1 amino acid sequence aligned with selected model organisms.

**Figure 6:**
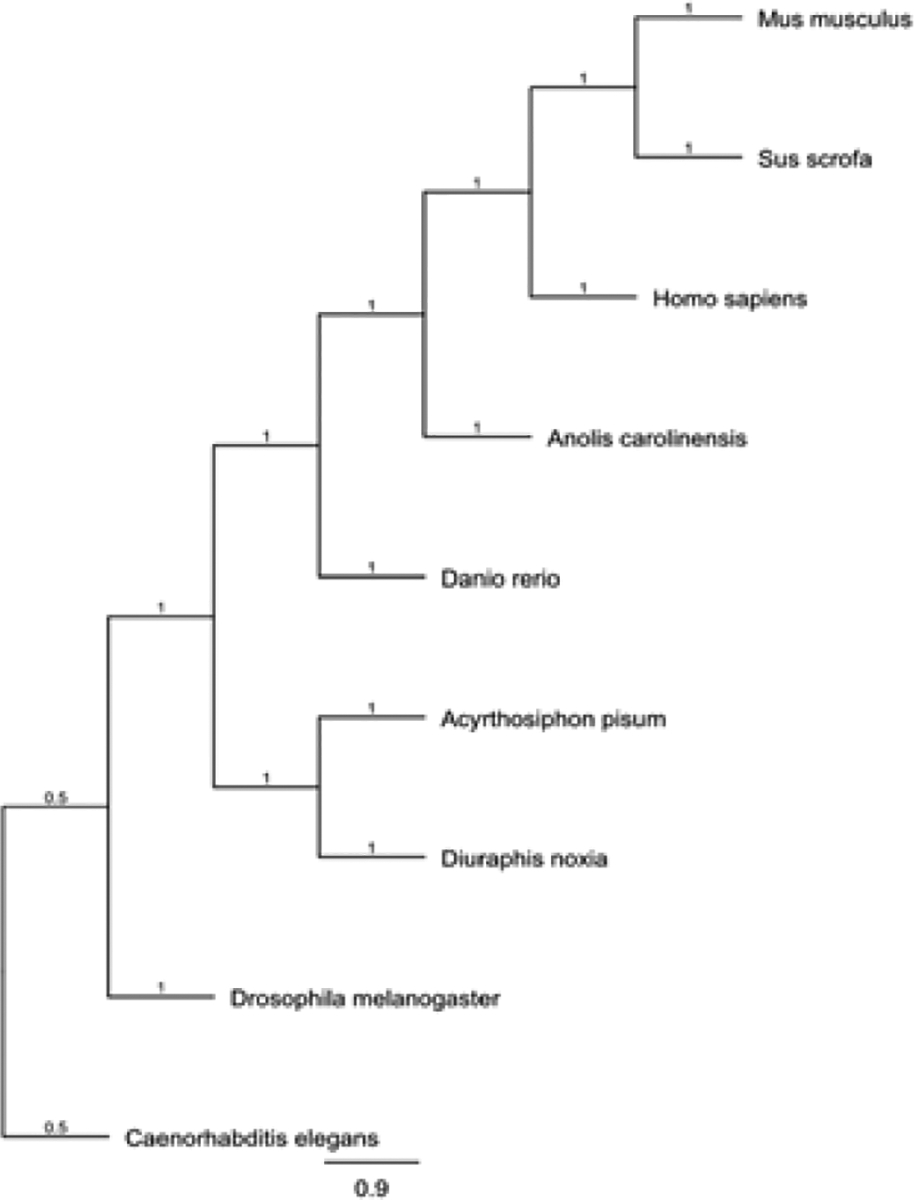
Neighbor-joining phylogenetic tree indicating the evolutionary relationship of pea aphid (*Acyrthosiphon pisum*) IRE1, and selected model organisms.

A multiple sequence alignment and phylogenetic tree of IRE1 generated using Genious software are indicated in Figures 5 and 6, respectively. Analysis with the NCBI conserved domain tool demonstrated the N-terminus contains a luminal kinase responsible for auto-phosphorylation of IRE1 dimers. The C-terminal contains both an ATP binding site and cytoplasmic RNase domain responsible for endonuclease activity of the gene. These IRE1 domains are conserved in other organisms also. The comparison of IRE1 sequences against the pea aphid reference genome sequence database available in GenBank demonstrated that the transcript aligned with an unplaced scaffold within the assembly (GenBank Accession Number: NW_003383494.1).

**Figure 7:**
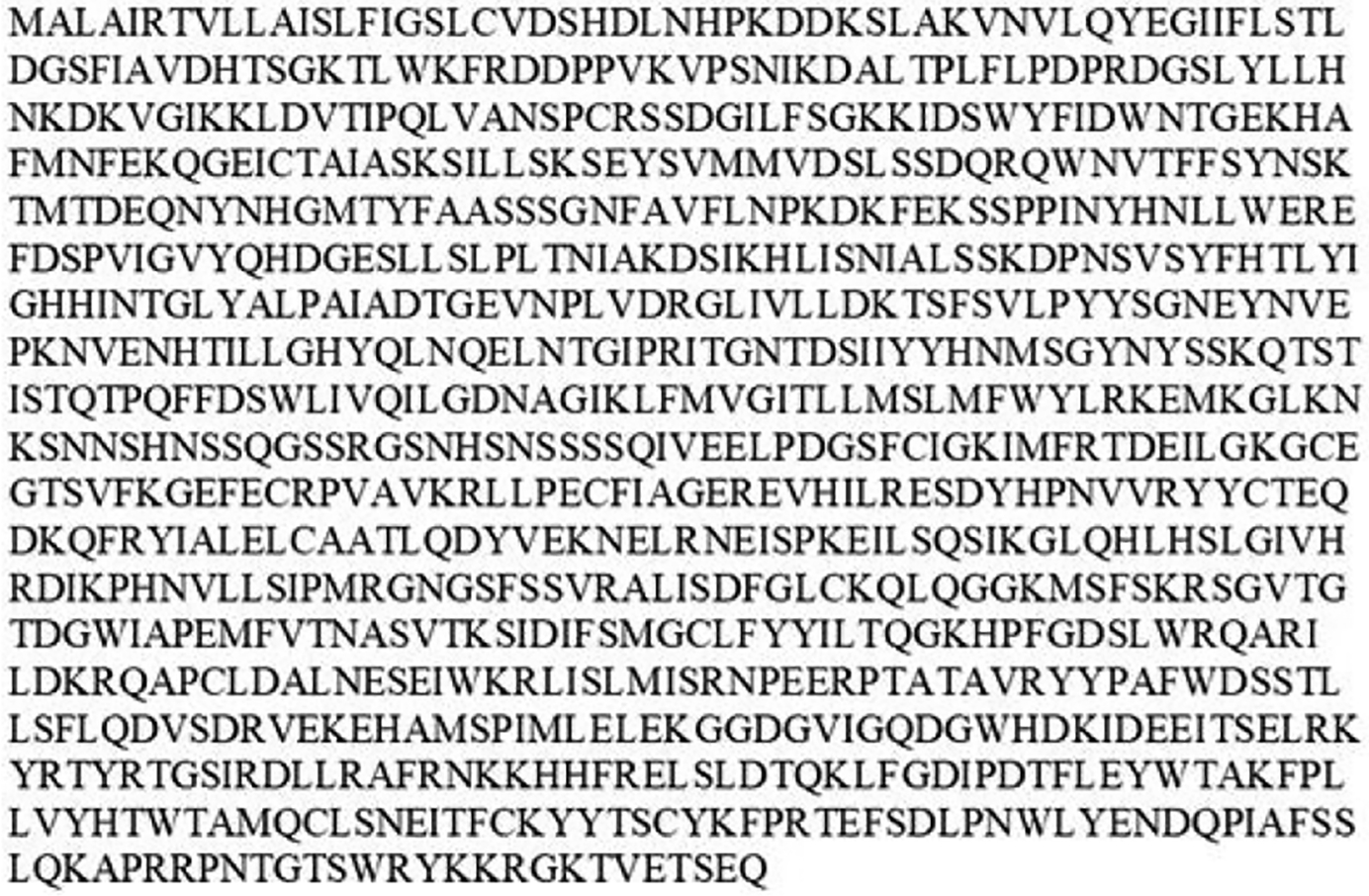
Predicted amino acid sequence of pea aphid PERK transcript variant X1 acquired using GenBank.

Screening of GenBank yielded two highly homologous PERK sequences found in pea aphids. Figure 7 shows the amino acid sequence of PERK isoform X1, which was chosen for this study.

**Figure 8:**
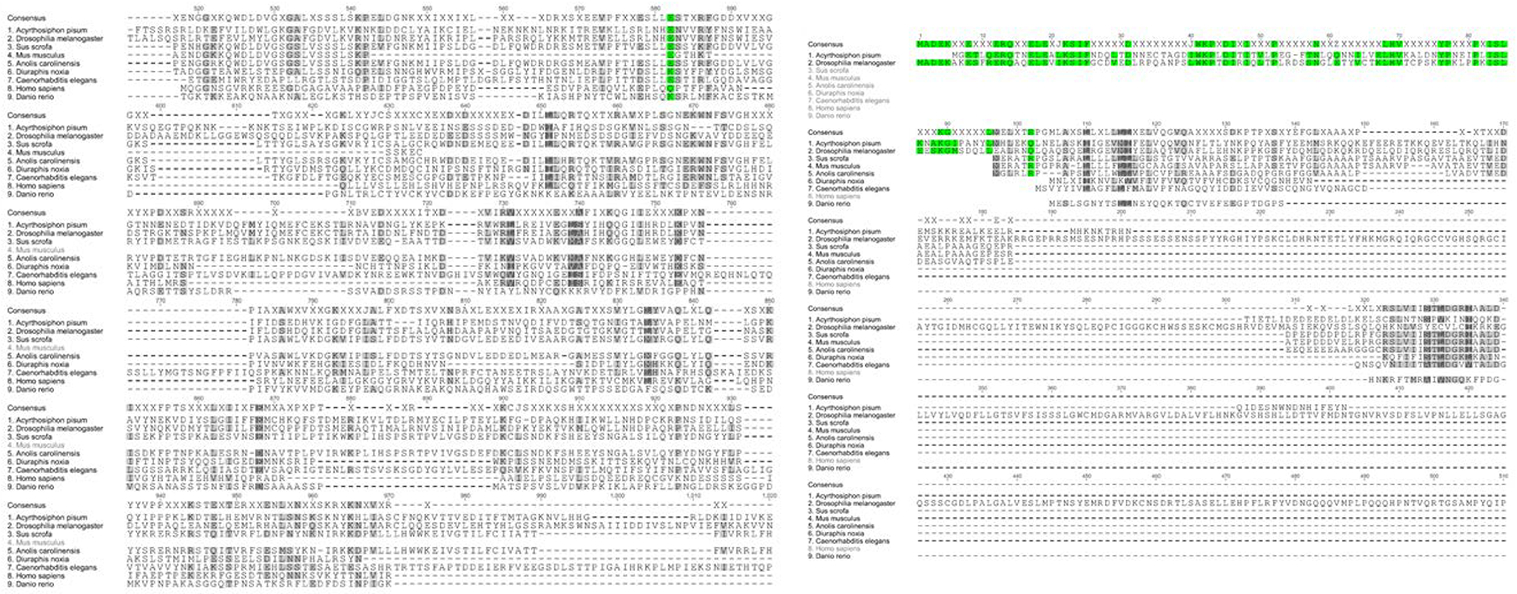

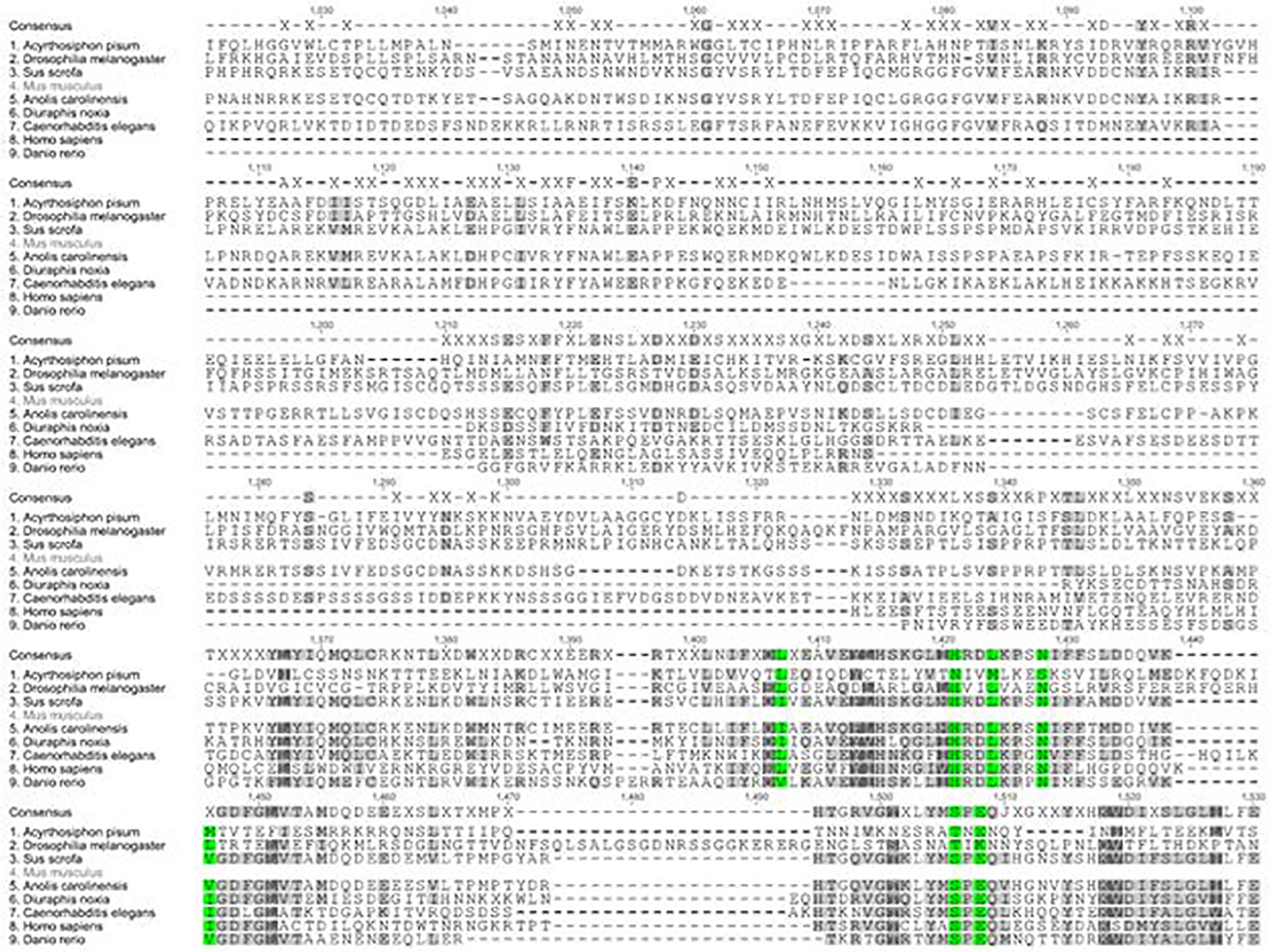
Multiple sequence alignment comparing pea aphid PERK amino acid sequence aligned with selected model organisms.

**Figure 9:**
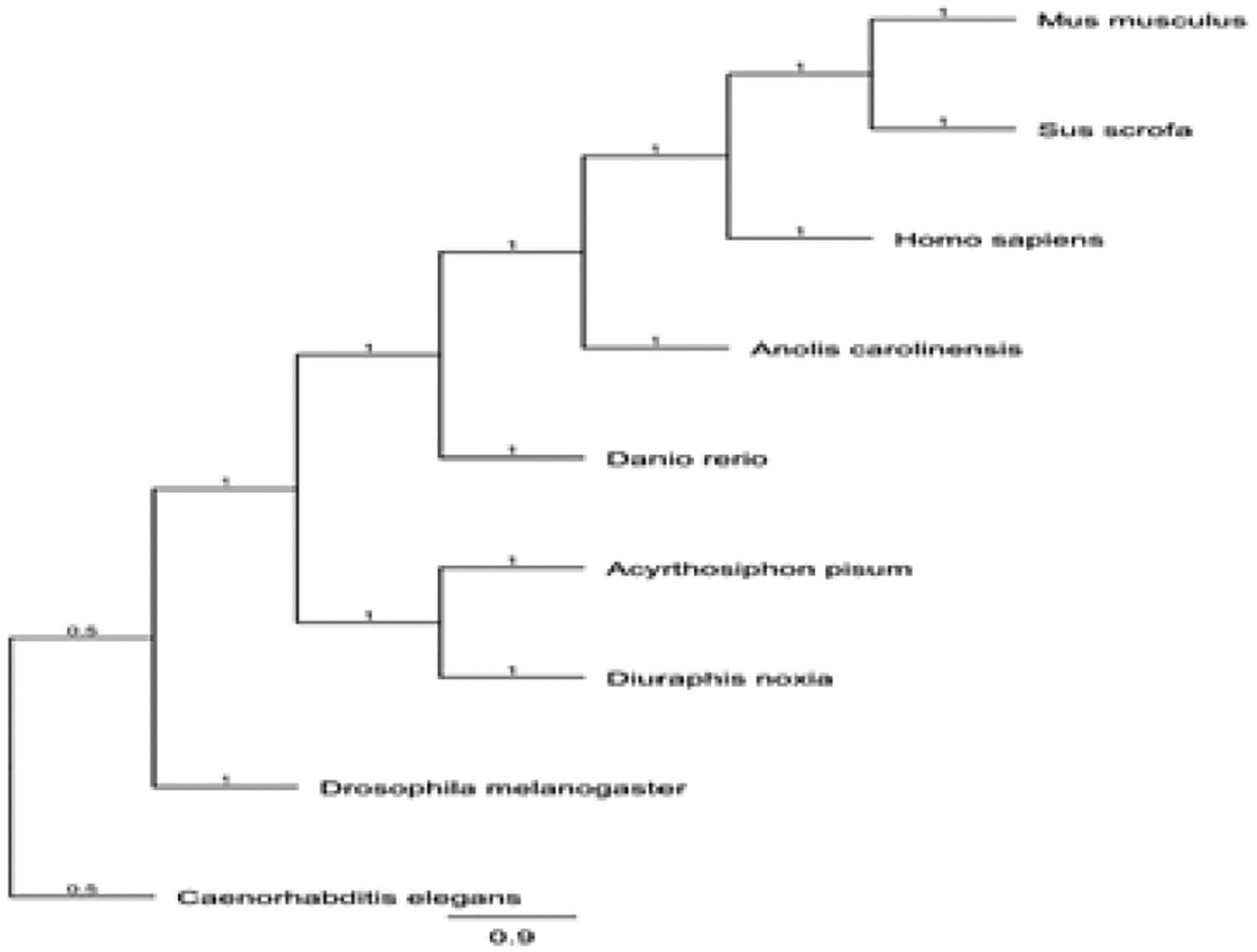
Neighbor-joining phylogenetic tree indicating the evolutionary relationship of pea aphid (*Acyrthosiphon pisum*) PERK and selected model organisms.

A multiple sequence alignment and phylogenetic tree of PERK generated using Genious software are indicated in Figures 8 and 9 respectively. Analysis with the NCBI conserved domain tool demonstrated the N-terminus contains a luminal kinase responsible for auto-phosphorylation of PERK dimers. The C-terminus contains the PERK catalytic domain which is responsible for the enzymatic activity of the gene. These domains of PERK are conserved in other organisms. The comparison of PERK sequences against the pea aphid reference genome sequence available in GenBank demonstrated that the transcript aligned with an unplaced scaffold with the assembly (GenBank Accession Number: NW_003383953.1).

### Effects of Variable dsRNA Concentration on Insect Lifespan

**Figure 10:**
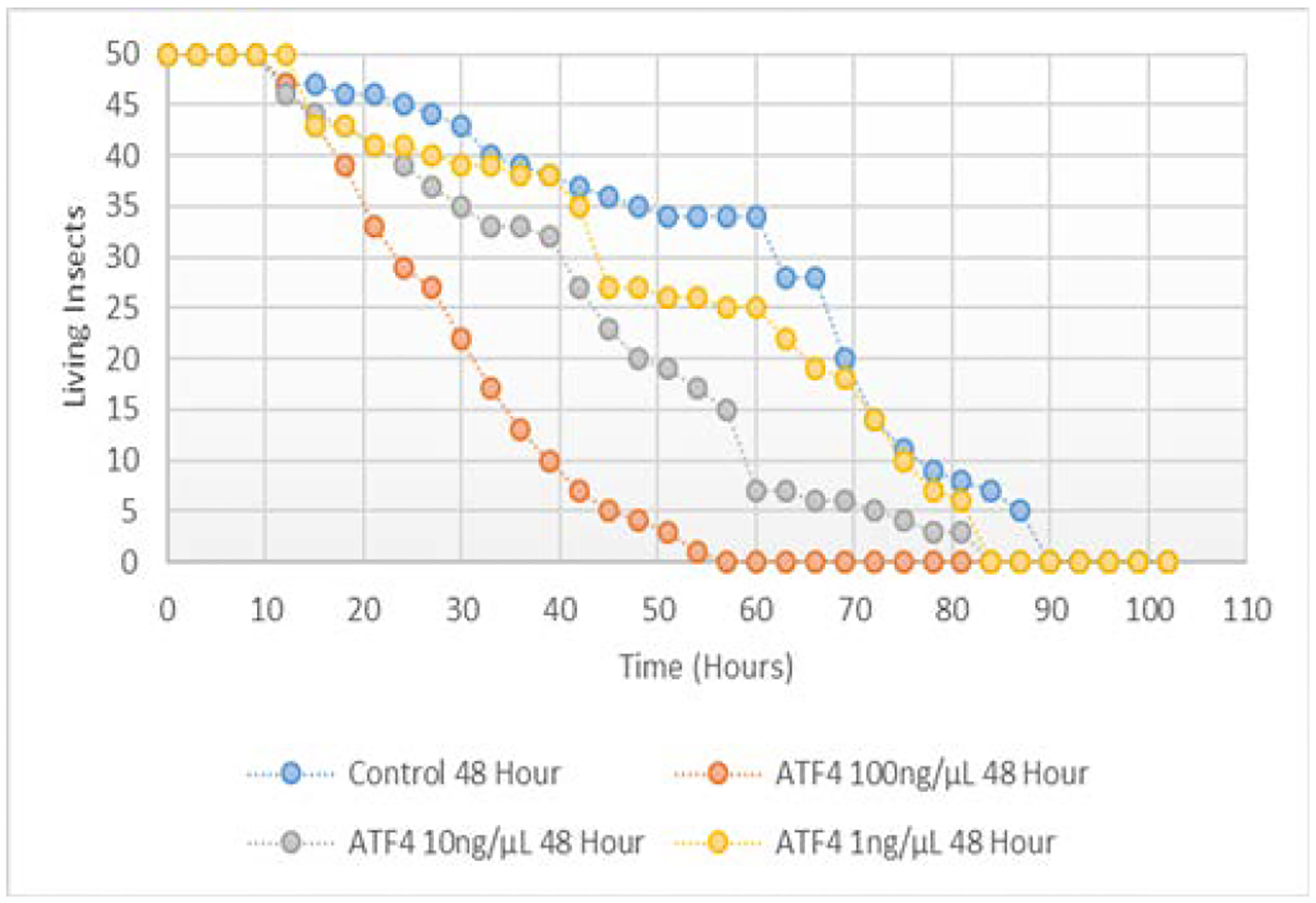
Survival in hours of pea aphids fed variable concentrations of ATF4 ds RNA.

All comparisons in this Section were made by a Mentel-Cox (log-rank) test. Difference in aphid survival was observed among treatments fed variable concentrations of ATF4 dsRNA (100 ng/μL, 10 ng/μL and 1 ng/μL) compared to the control (Figure 10). Survival in hours of the 100 ng/μL treatment group was significantly less than the survival in hours of the control group (p<0.05). Survival in hours of the 10 ng/μL tended to be lower than the survival in hours of the control group (p<0.10). Survival in hours of the 1 ng/μL was not statistically different from the survival in hours of the control group (p>0.10).

**Figure 11:**
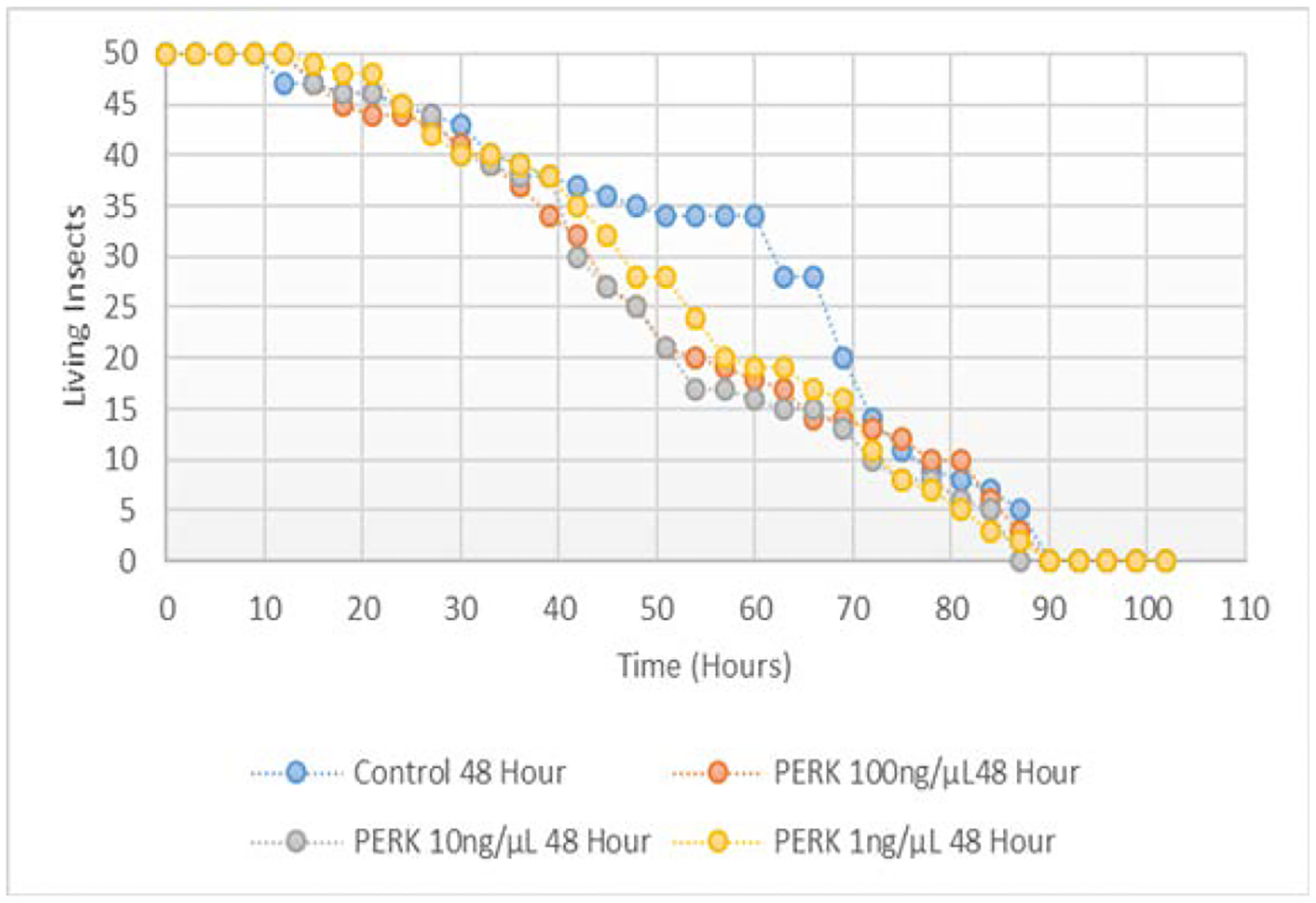
Survival in hours of pea aphids fed variable concentrations of ATF4 ds RNA.

**Figure 12:**
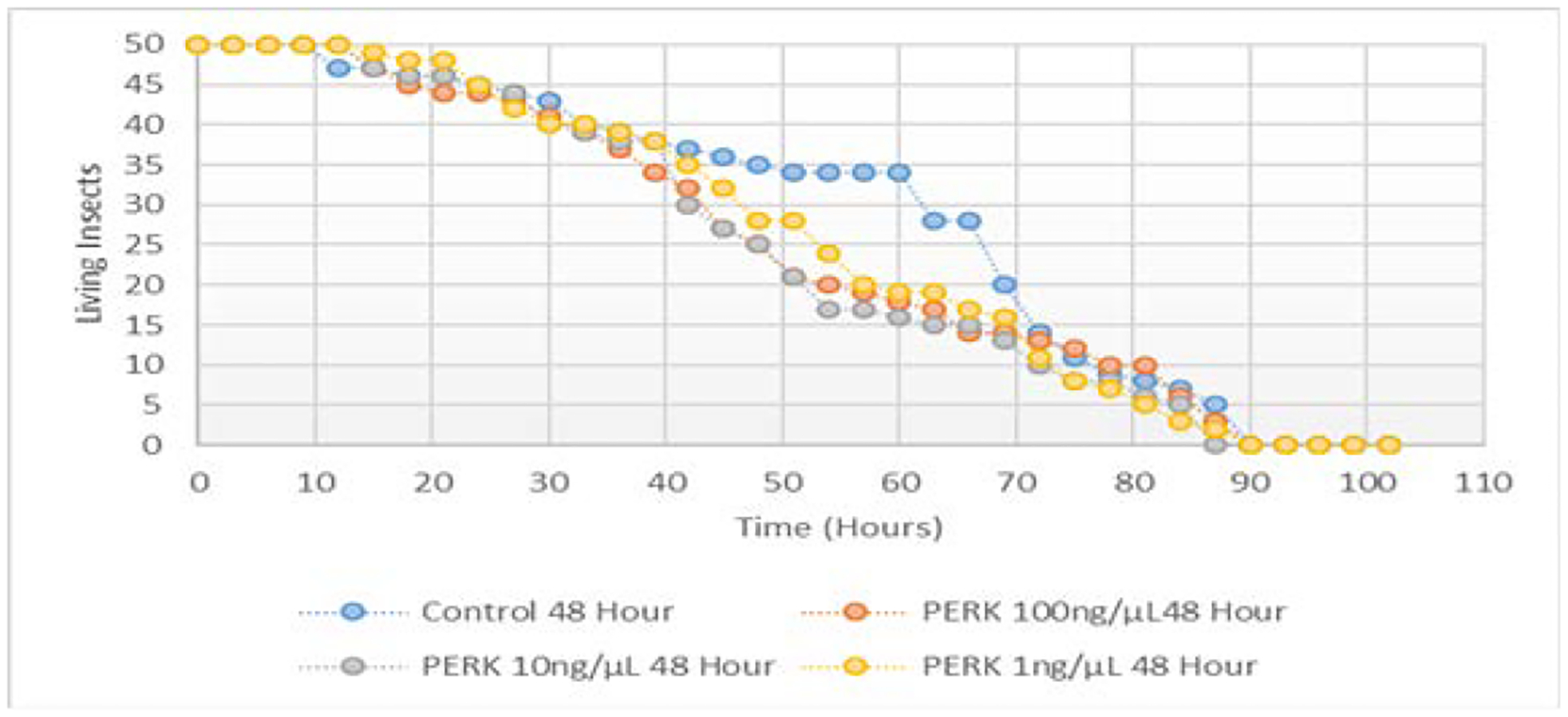
Survival in hours of pea aphids fed variable concentrations of PERK ds RNA.

No difference in aphid survival was observed when fed variable concentrations of both IRE1 and PERK dsRNA (100 ng/μL, 10 ng/μL, and 1 ng/μL) when compared to the control population are indicated in Figures 11 and 12 respectively. Survival in hours of all treatments was not statistically different from the survival in hours of the control group (p>0.10).

### Real-Time qRT-PCR

**Figure 13:**
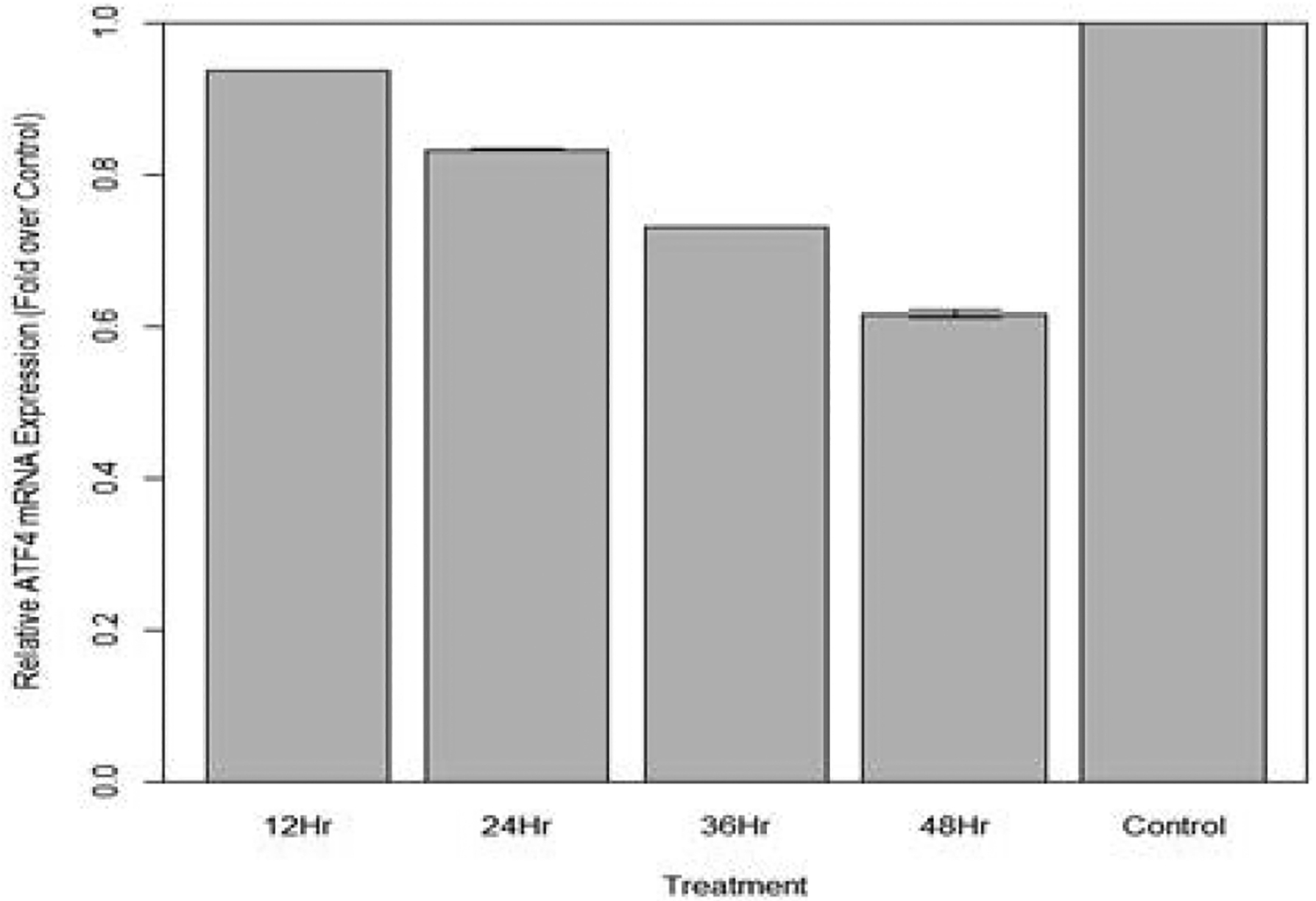
Relative expression of total ATF4 in aphids fed artificial diet containing 100 ng/μL ATF4 dsRNA over a 48-hour period (relative expression ± SD; n=10 aphids per dish, 3 dishes per treatment, P<0.05).

A One Way Analysis of Variance (ANOVA) followed by a Tukey’s test was used to provide statistical inference. Changes in relative expression of ATF4 in aphids fed diet containing 100 ng/μL ATF4 dsRNA are indicated in Figure 13. Change in expression over 12 hours was not statistically significant when compared to the control (p>0.10). However, after 24 hours, expression of ATF4 was significantly lower in fed aphids than in the control (p<0.05), a mean decrease in expression by 6.22% was observed. At 36 hours, expression was significantly lower in fed aphids than in the control (p<0.05): A mean decrease in expression by 16.7% was observed. At 48 hours expression was significantly lower in fed aphids than in the control (p<0.05): A mean decrease in expression of ATF4 by 39.3% was observed.

**Figure 14:**
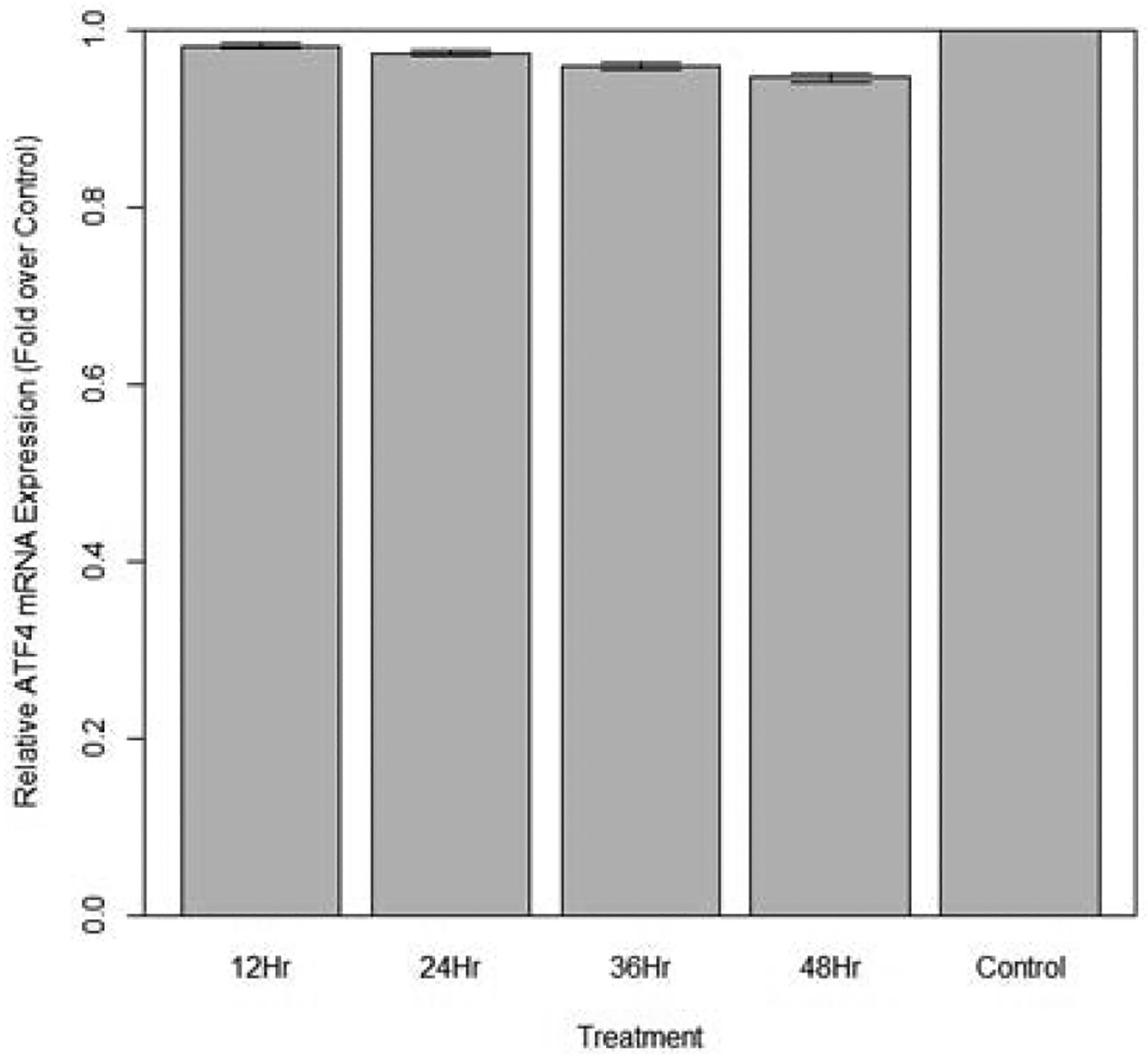
Relative expression of total ATF4 in aphids fed artificial diet containing 10 ng/μL ATF4 dsRNA over a 48-hour period (relative expression ± SD; n=10 aphids per dish, 3 dishes per treatment, P<0.05).

Changes in relative expression of ATF4 in aphids fed diet containing 10 ng/μL ATF4 dsRNA are indicated in Figure 14. Change in expression over 12, 24 and 36 hours was not statistically significant when compared to the control (p>0.10). At 48 hours, expression of ATF4 was significantly lower in fed aphids than in the control (p<0.05): Mean decrease in expression of by 5.17% was observed.

**Figure 15:**
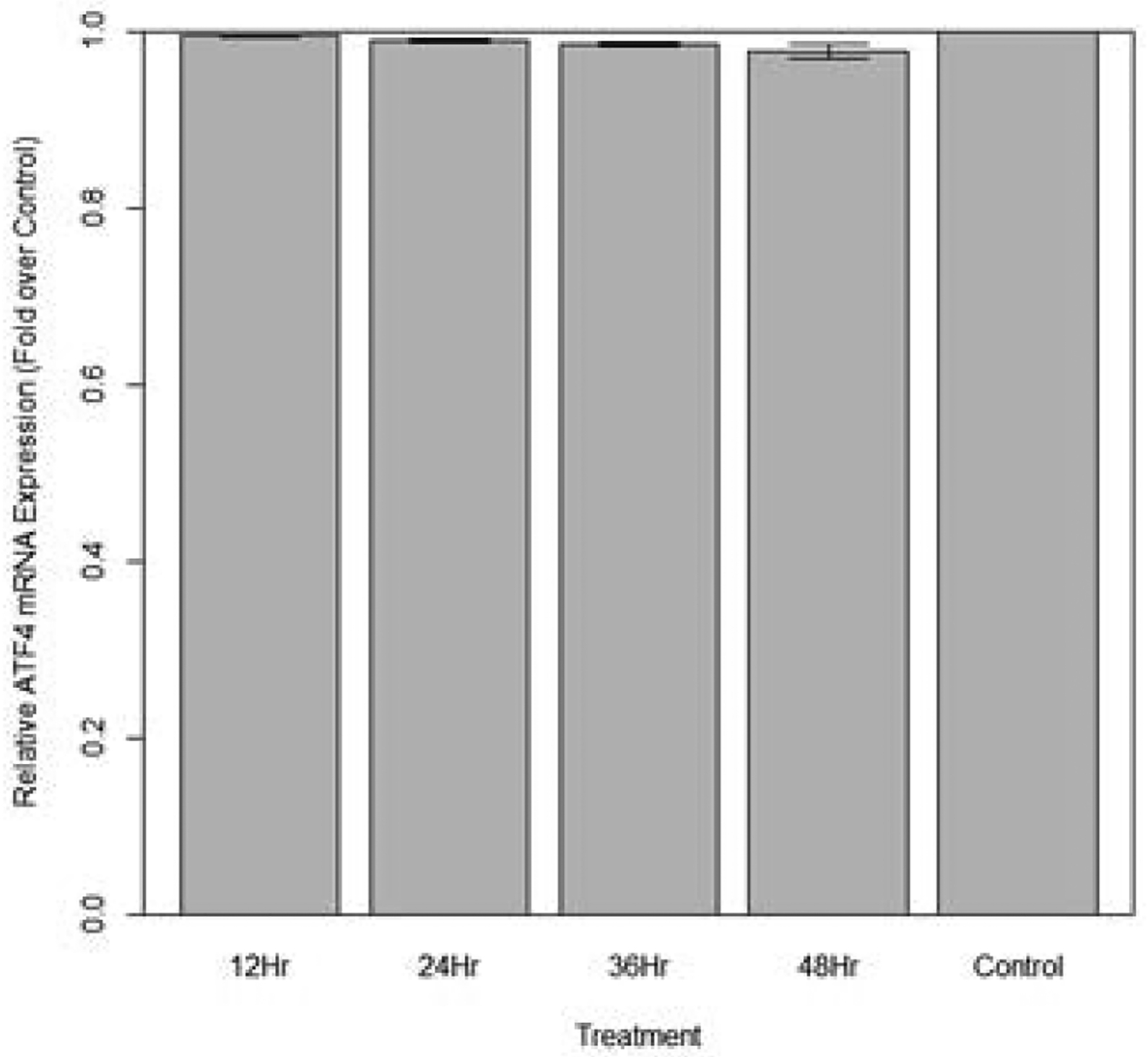
Relative expression of total ATF4 in aphids fed artificial diet containing 1 ng/μL ATF4 dsRNA over a 48-hour period (relative expression ± SD; n=10 aphids per dish, 3 dishes per treatment, P<0.05).

Changes in expression of ATF4 in aphids fed diet containing 1 ng/μL ATF4 dsRNA are indicated in Figure 15. No significant change in ATF4 expression was observed (p>0.10) over the course of the treatment.

**Figure 16:**
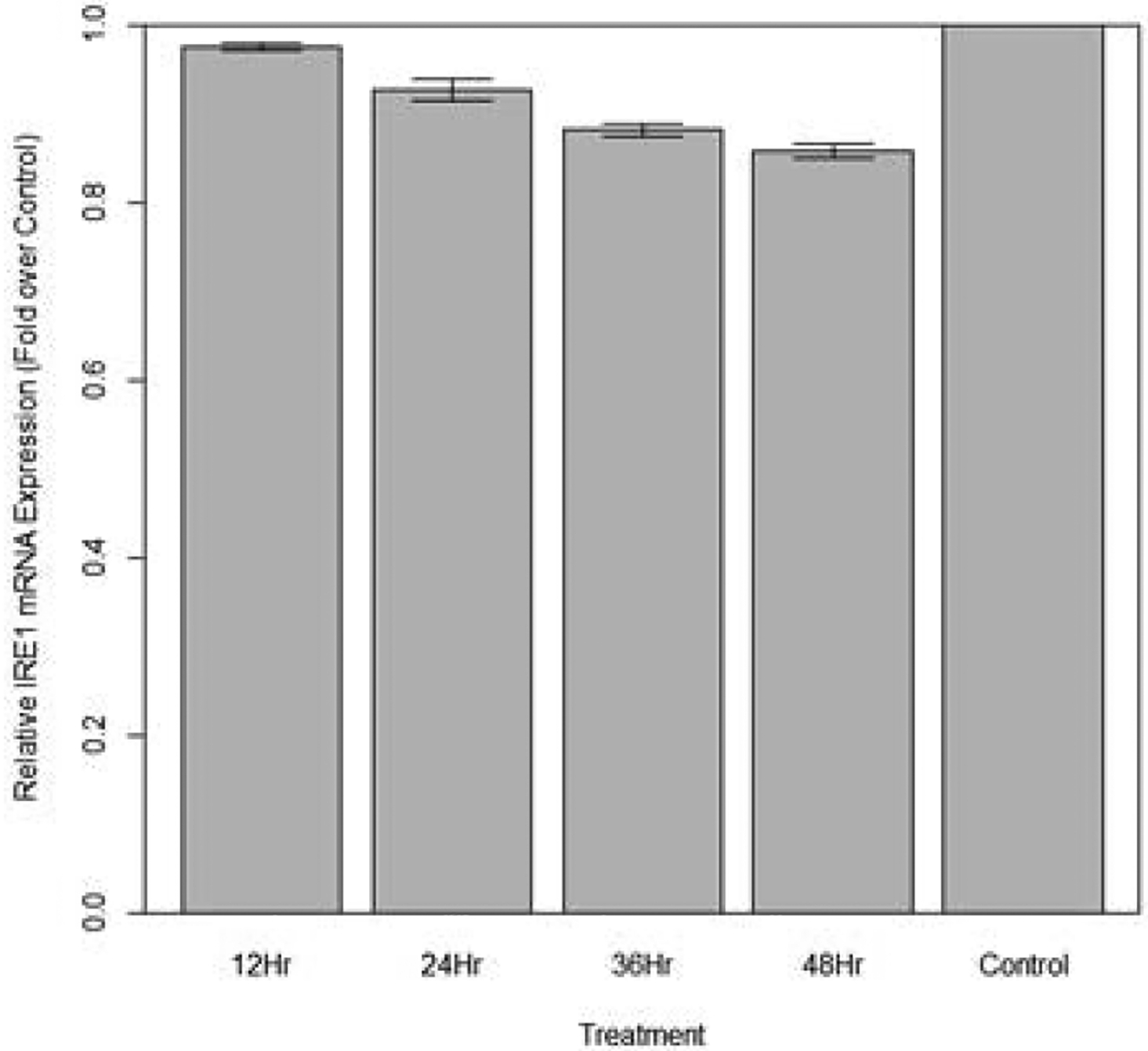
Relative expression of total IRE1 in aphids fed artificial diet containing 100 ng/μL IRE1 dsRNA over a 48-hour period (relative expression ± SD; n=10 aphids per dish, 3 dishes per treatment, P<0.05).

Changes in relative expression of IRE1 in aphids fed diet containing 100 ng/μL IRE1 dsRNA are indicated in Figure 16. The expression of IRE1 over 12, 24, 36, and 48 hours were significantly lower in fed aphids when compared to the control (p<0.05): Mean decreases in expression of IRE1 by 9.23%, 16.4%, 22.5% and 37.4% respectively were observed.

**Figure 17:**
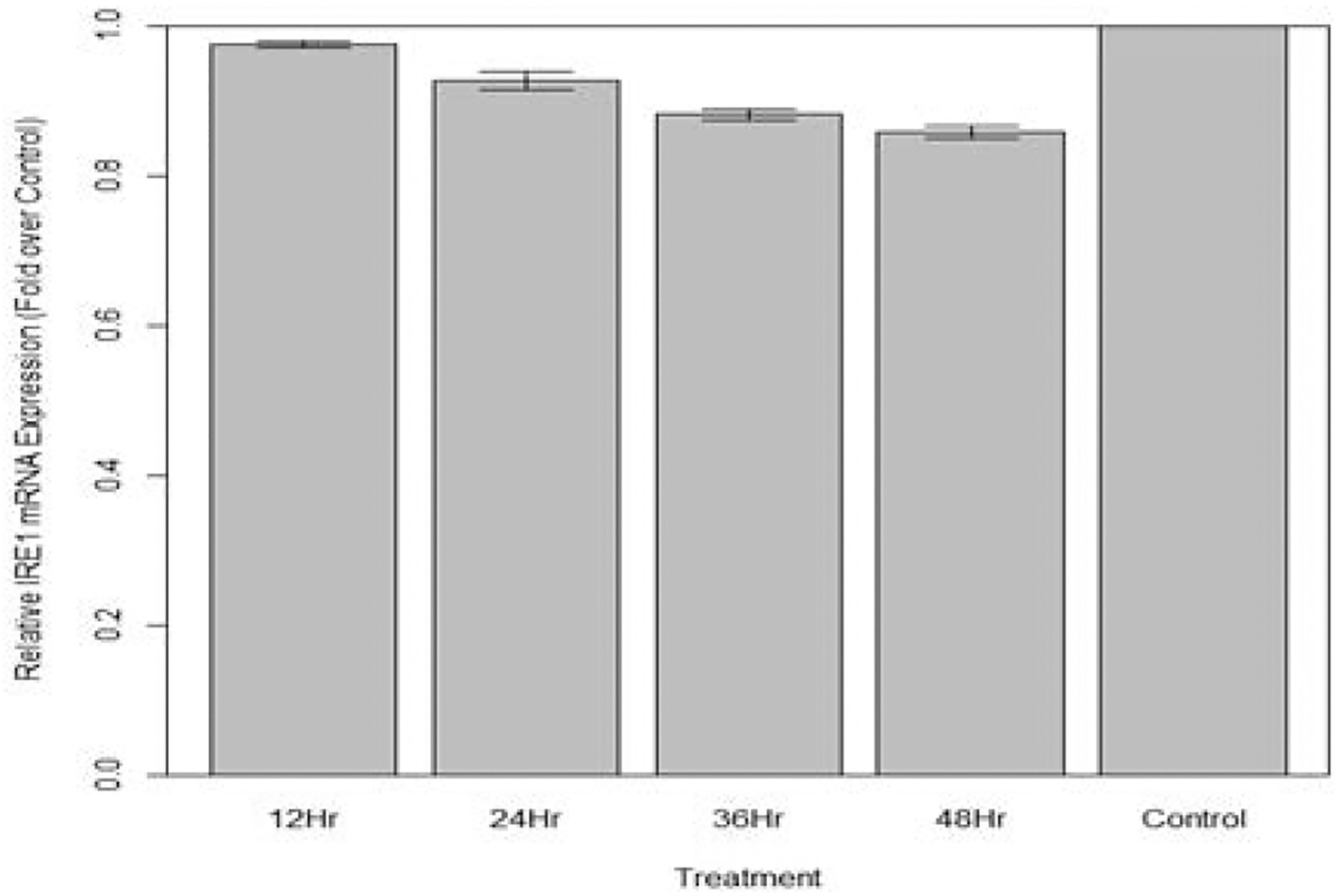
Relative expression of total IRE1 in aphids fed artificial diet containing 10 ng/μL IRE1 dsRNA over a 48-hour period (relative expression ± SD; n=10 aphids per dish, 3 dishes per treatment, P<0.05).

Changes in relative expression of IRE1 in aphids fed diet containing 10 ng/μL IRE1 dsRNA are indicated in Figure 17. The expression of IRE1 over 12 hours was not statistically significant when compared to the control (p>0.10). The expression of IRE1 over 24, 36, and 48 hours was significantly lower in fed aphids when compared to the control (p<0.05): Mean decreases in expression of 7.2%, 11.7%, 13.2% respectively were observed.

**Figure 18:**
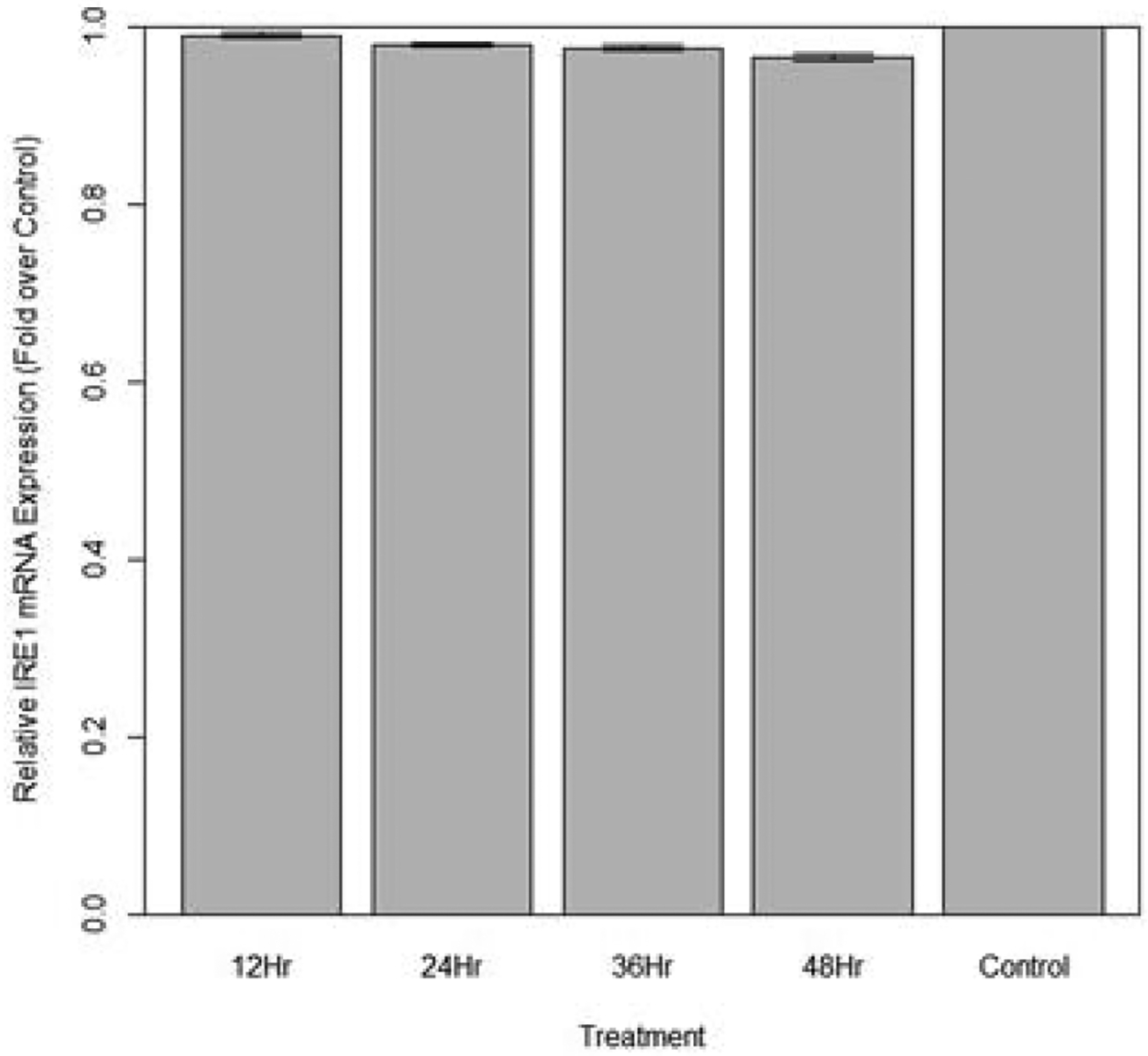
Relative expression of total IRE1 in aphids fed artificial diet containing 1 ng/μL IRE1 dsRNA over a 48-hour period (relative expression ± SD; n=10 aphids per dish, 3 dishes per treatment, P<0.05).

Changes in relative expression of IRE1 in aphids fed diet containing 1 ng/μL IRE1 dsRNA are indicated in Figure 18. No significant change in IRE1 expression was observed (p>0.10) over the course of the treatment.

**Figure 19:**
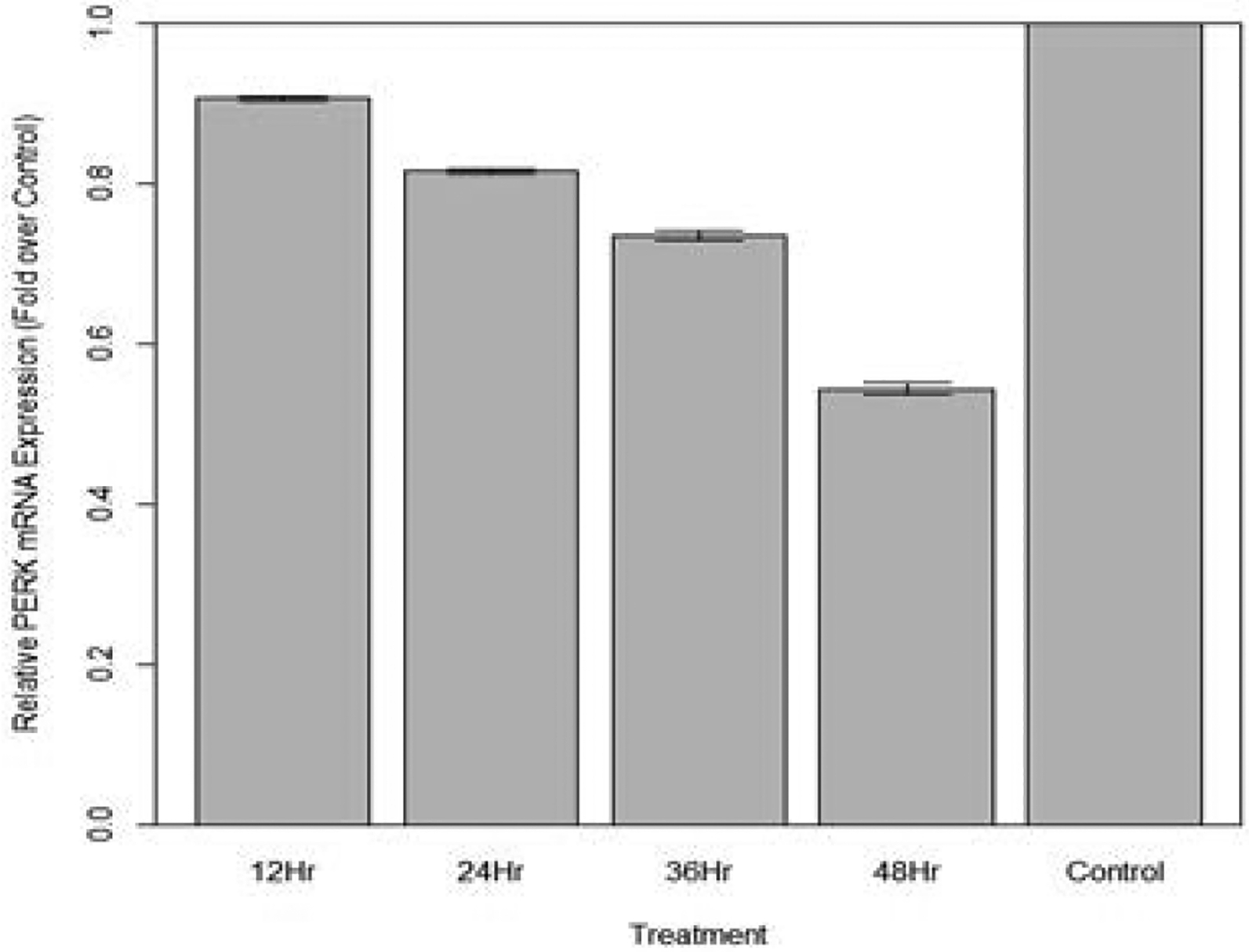
Relative expression of total PERK in aphids fed artificial diet containing 100 ng/μL PERK dsRNA over a 48-hour period (relative expression ± SD; n=10 aphids per dish, 3 dishes per treatment, P<0.05).

Changes in relative expression of PERK in aphids fed diet containing 100 ng/μL PERK dsRNA are indicated in Figure 19. The expression of PERK over 12, 24, 36, and 48 hours was significantly lower in fed aphids when compared to the control (p<0.05): Mean decreases in expression of by 11.4%, 19.3%, 27.5%, and 46.2% respectively were observed.

Changes in relative expression of PERK in aphids fed diet containing 10 ng/μL PERK dsRNA are indicated in. The expression of IRE1 over 12 hours was not statistically significant when compared to the control (p>0.10). The expression of PERK over 24, 36, and 48 hours was significantly lower in fed aphids when compared to the control (p<0.05): Mean decreases in expression of 10.4%, 11.7%, and 15.6 respectively were observed.

**Figure 20:**
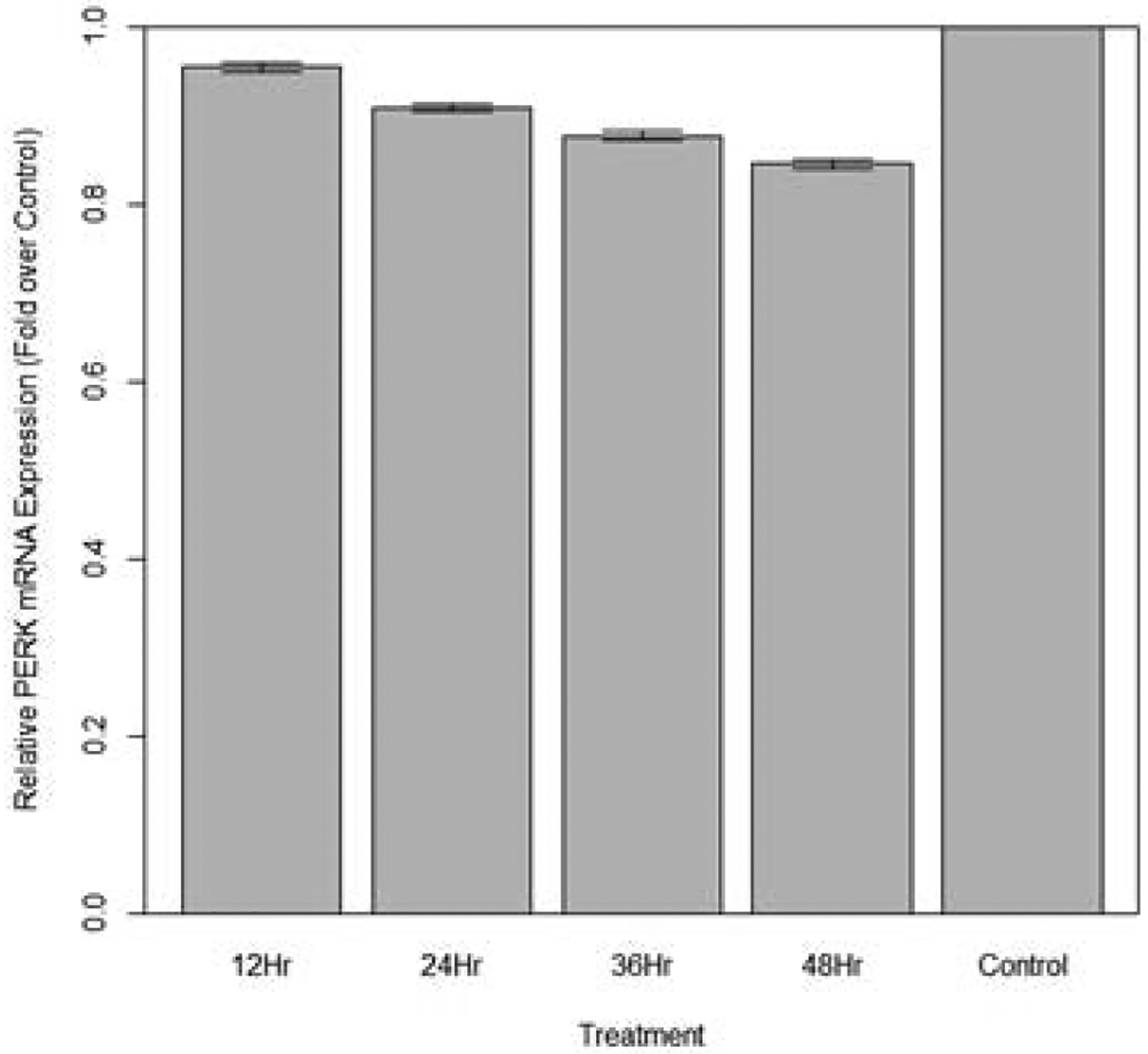
Relative expression of total PERK in aphids fed artificial diet containing 10 ng/μL PERK dsRNA over a 48-hour period (relative expression ± SD; n=10 aphids per dish, 3 dishes per treatment, P<0.05).

**Figure 21:**
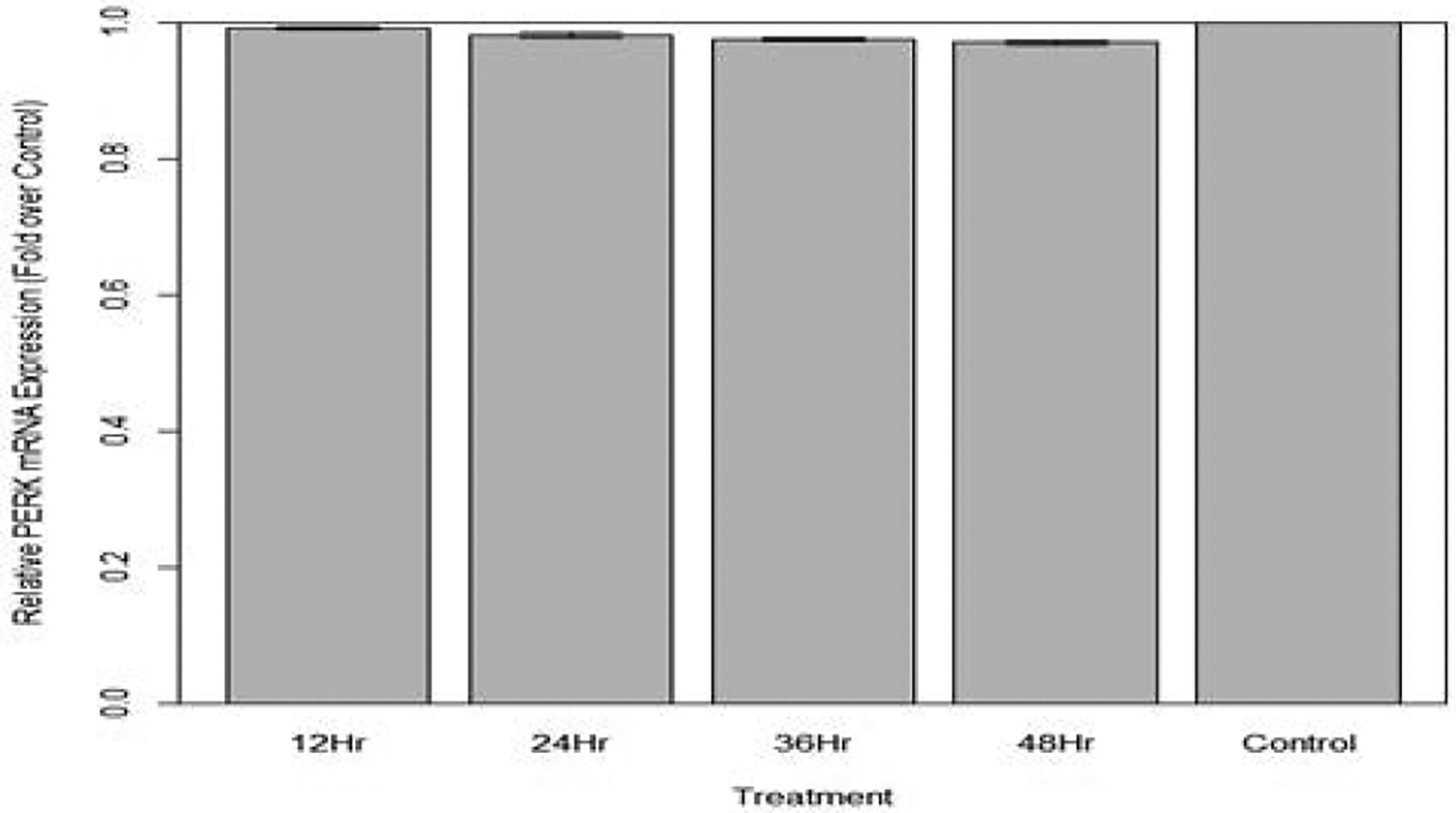
Relative expression of total PERK in aphids fed artificial diet containing 1 ng/μL PERK dsRNA over a 48-hour period (relative expression ± SD; n=10 aphids per.

Changes in relative expression of PERK in aphids fed diet containing 1 ng/μL PERK dsRNA are indicated. No significant change in PERK expression was observed (p>0.10) over the course of the treatment (Figures 20 and 21).

## DISCUSSION

Previous studies [13,15] have indicated that Activating Transcription Factor 4 (ATF4) participates in an adaptive role in cellular processes in the form of multiple transcription site promotion and repression. The evolution of the ATF4 gene appears to be highly conserved among the examined model organisms. In humans, the ATF4 sequence is located on chromosome 22 [13], however, in pea aphids, the ATF4 sequence has not been mapped to a specific chromosome.

Although the location of the ATF4 sequence within the genome differs among species, the predicted ATF4 sequence of pea aphids is highly similar to the predicted amino acid sequences of the examined model organisms. These results suggest that the ATF4 sequence has been highly conserved throughout the evolutionary process, which indicates the functional importance of the gene in vertebrates and invertebrates. In mammals, ATF4 contains the highly conserved bZIP region necessary for DNA binding. The analysis of predicted pea aphid ATF4 indicates that aphid ATF4 also contains the conserved bZIP region. Additionally, the amino acid sequence of pea aphid ATF4 found in this region was highly homologous to mammalian ATF4. The results of this study indicated that ATF4 in both vertebrates and invertebrates appears to be highly conserved in its genome structure, as well as amino acid sequence. Furthermore, domain analysis demonstrated a high degree of conservation in the bZIP domain found in ATF4, suggesting that ATF4 is highly conserved among all examined organisms. Given the high degree of genetic and structural conservation observed between pea aphid ATF4 and those of other species, it is concluded that ATF4 may not be an ideal target for knockdown by means of RNAi due to possible off-target effects of anti-ATF4 dsRNA.

Previous studies have indicated that Inositol requiring Enzyme 1 (IRE1) activation affects the overall rate of protein synthesis, and determines cell fate under stress [6,19]. The evolution of the IRE1 gene appears to be highly conserved among the examined model organisms. In humans, the IRE1 sequence is located on chromosome 17 [17] however, in pea aphids, the IRE1 sequence has not been mapped to a specific chromosome.

Although the location of the IRE1 sequence within the genome differs among species, the predicted IRE1 sequence of pea aphids is similar to the predicted amino acid sequences of the examined model organisms. The results suggest that the IRE1 sequence has been conserved throughout the evolutionary process, which indicates the functional importance of the gene in vertebrates and invertebrates. In mammals, IRE1 contains the conserved endonuclease domain responsible for ER stress response. The analysis of predicted pea aphid IRE1 indicates that aphid IRE1 also contains the conserved endonuclease domain. Additionally, the amino acid sequence of pea aphid IRE1 found in this region was highly homologous to mammalian IRE1. The results of this study indicate that IRE1 in both vertebrates and invertebrates appears to be conserved in its genome structure, as well as amino acid sequence. Compared to ATF4, IRE1 is a much larger protein. As such, it is easier to identify sequences of the predicted mRNA to target while minimizing possible off target effects during RNAi knockdown.

Previous studies have indicated that Eukaryotic Initiation Factor-2-alpha Kinase (PERK) activation affects overall rate of protein synthesis and determines cell fate under stress [8,9]. The evolution of the PERK gene appears to be highly conserved among the examined model organisms. In humans, the PERK sequence is located on chromosome 2 [9]. However, in pea aphids, the PERK sequence has not been mapped to a specific chromosome.

Although the location of the PERK sequence within the genome differs among species, the predicted PERK sequence of pea aphids is dissimilar to the predicted amino acid sequences of the examined model organisms. These results suggest that the PERK sequence has been loosely conserved throughout the evolutionary process, which indicates the functional importance of the gene in vertebrates and invertebrates. In mammals, PERK contains a conserved N-terminus kinase domain. The analysis of predicted pea aphid PERK indicates that it also contains the conserved kinase domain. Additionally, the amino acid sequence of pea aphid PERK found in this region was highly homologous to mammalian PERK. The results of this study indicated that PERK in both vertebrates and invertebrates appears to be loosely conserved in its genome structure, as well as amino acid sequence. Compared to ATF4, PERK is a much larger protein. As such, it is easier to identify sequences of the predicted mRNA to target while minimizing possible off-target effects during RNAi knockdown.

Although other organisms’ ATF4 sequence must be carefully considered, knockdown of ATF4 mRNA shows promise as an effective means of aphid population control through ingested ATF4 dsRNA. Aphids fed artificial diet containing 100 ng/μL ATF4 dsRNA exhibited significantly decreased survival (t1/2=27 hours) compared to aphids fed artificial diet that did not contain dsRNA (t1/2=67 hours). This result is supported by the qPCR analysis of aphids fed ATF4 dsRNA at this concentration. By hour 24, a mean 6.22% decrease in ATF4 mRNA was observed. As the observed knockdown of ATF4 continued, by hour 48, a mean 39.3% decrease in ATF4 mRNA expression was observed; coinciding with the accelerated rate of death of aphids fed dsRNA at this concentration (mean surviving aphids at 48 hours=3). Knockdown of ATF4 with a diet containing 10 ng/μL of ATF4 dsRNA has been achieved; however, the rate of knockdown was slower compared to its undiluted form: By hour 48, a mean 5.17% decrease in ATF4 mRNA was observed. Survival rate of aphids fed 10 ng/μL ATF4 dsRNA was still much higher compared to the undiluted dose (t1/2=42 hours). No significant change in survival or mRNA expression was observed in the aphids fed 1 ng/μL ATF4 dsRNA.

Successful knockdown of IRE1 mRNA was achieved in pea aphids through the feeding of 10 and 100 ng/μL IRE1 dsRNA resulting in a mean decrease of 13.2% and 37.4% in expression, respectively, over 48 hours. This knockdown did not coincide with a decreased survival of aphids treated (t1/2=45 hours) when compared to the control (t1/2=67 hours).

Successful knockdown of PERK mRNA was achieved in pea aphids through the feeding of 10 and 100 ng/μL PERK dsRNA resulting in a mean decrease of 15.6% and 46.2% in expression, respectively, over 48 hours. This knockdown did not coincide with a decreased survival of aphids treated (t1/2=48 hours) when compared to the control (t1/2=67 hours).

The successful knockdown of the UPR genes indicate that enough dsRNA must be fed to overwhelm the rate of transcription of the target mRNA.

A previous study has determined that ATF4 modulates the transcription of genes involved in amino acid biosynthesis and catabolism, as well as genes of the UPR. One of the primary enzymes regulated by ATF4 is asparagine synthetase [16]. The results of this study suggest that ATF4 is active in cellular function without activation of the UPR. It is also suggested that expression of ATF4 mRNA is highly sensitive to introduction of ATF4 dsRNA. Because ATF4 is critical for proper cellular function, reduction in expression of ATF4 is fatal in pea aphids.

Introduction of dsRNA complementary to IRE1 and PERK, respectively, did not cause a change in pea aphid survival. IRE1 and PERK are activated by the accumulation of unfolded protein within the ER lumen, and function as signal transducers of the UPR [12]. Redundancy of function is a common phenomenon in living systems. Because IRE1 and PERK share the ultimate function of relieving ER stress, it is possible that knockdown of IRE1 does not affect aphid survival because ATF6 and PERK might be able to maintain UPR function without the action of IRE1. This is also the case in the knockdown of PERK; ATF6 and IRE1 might be able to maintain UPR function without the action of PERK.

The 100 ng/μL dose of each dsRNA caused a decrease in mean expression of 39.3%, 37.4% and 46.2% of ATF4, IRE1, and PERK mRNAs, respectively. A higher concentration of dsRNA may be more successful in decreasing expression of the target genes mRNAs and remains to be explored.

The results of this study agree with previous RNAi experiments in pea aphids [24,25], where dsRNA complementary to an mRNA was introduced to the aphid diet, and insect survival was affected negatively. In the study published by [22], the target gene encoded for a protein produced in the salivary glands of aphids. The knockdown of this protein prevented aphids from properly feeding. Because this gene and its homologs are only expressed in phloem-feeding insects, this mitigates many of the possible off-target effects associated with RNAi. This method of choosing targets that are specific to the target organism is the preferred method of engineering lethal dsRNAs.

To evaluate if aphid survival can be affected by knockdown of the ER transmembrane signal transducers, a cocktail of ATF6, IRE1 and PERK dsRNAs could be delivered to pea aphids. This may cause a decrease in survival by eliminating redundant function.

## CONCLUSION

In summary, this study was the first to explore knockdown of ATF4, IRE1, and PERK and knockdown impact on aphid survival. The predicted amino acid sequences of the pea aphid genes investigated shared a high degree of sequence similarity with the ATF4, IRE1, and PERK of the model organisms investigated. The feeding of ATF4 dsRNA caused significant decrease in pea aphid survival, while feeding of IRE1 and PERK dsRNAs did not cause significant decrease survival. The exact mechanisms involved in RNAi mediated knockdown of ATF4, IRE, and PERK remains unknown.

## Figures and Tables

**Table 1:** List of primers used for dsRNA synthesis and qRT-PCR.

Primer	GenBank Accession#	Sequence
ATF4 dsRNA sense	This manuscript	TAATACGACTCACTATAGGGACGGCGAGTGCCAATATG
ATF4 dsRNA antisense	This manuscript	TAATACGACTCACTATAGGGAATCTTCTTTCTCGTCAACAACC
IRE1 dsRNA sense	This manuscript	TAATACGACTCACTATAGGGTGCGCTGAAATTCTGTTTACTGT
IRE1 dsRNA antisense	This manuscript	TAATACGACTCACTATAGGGGGCCAATGCCATTTTGTCGT
PERK dsRNA sense	This manuscript	TAATACGACTCACTATAGGGCCAATACCATAGCGAAACAATA
PERK dsRNA antisense	This manuscript	TAATACGACTCACTATAGGGATAACAAAGCGATACCATAACC
ATF4 qPCR sense	This manuscript	CACTTATGACCCCGTAAGCC
ATF4 qPCR antisense	This manuscript	GGAAGCCATATTGGCACTCG
IRE1 qPCR sense	This manuscript	CATTATTACAAAAAGGTGTTCAGCG
IRE1 qPCR antisense	This manuscript	CCAGACGAGATGGTGGTAGC
PERK qPCR sense	This manuscript	TGTCCGAGCATCAGACACAC
PERK qPCR antisense	This manuscript	TGGGAGACTCCGATTTGTGAG
RPL27 qPCR sense	Avila, et al., 2018	TCGTTACCCTCGGAAAGTC
RPL27 qPCR antisense	Avila, et al., 2018	GTTGGCATAAGGTGGTTGT

## References

[1] Crick (1970) Central dogma of molecular biology. Nature 227: 561–563. 4913914 10.1038/227561a0

[2] Washburn S, Gottesman ME (2015) Regulation of transcription enlongation and termination. Biomolecules 5: 1063–1078. 26035374 10.3390/biom5021063 PMC4496710

[3] Käll L, Krogh A, Sonnhammer E, Erik LL (2004) A combined transmembrane topology and signal peptide prediction method. J Mol Biol 338: 1027–1036. 15111065 10.1016/j.jmb.2004.03.016

[4] Lodish H, Berk A, Zipursky SL (2000) Post-translational modifications and quality control in the rough ER. Mol Cell Biol 112: 367–381.

[5] Hetz C (2012) The unfolded protein response: Controlling cell fate decisions under ER stress and beyond. Nat Rev Mol Cell Biol 13: 89–96. 22251901 10.1038/nrm3270

[6] Walter P, Ron D (2011) The unfolded protein response: From stress pathway to homoestatic regulation. Science 334: 1081–1086. 22116877 10.1126/science.1209038

[7] Ron D, Walter P (2007) Signal integration in the endoplasmic reticulum unfolded protein response. Nat Rev Mol Cell Biol 7: 519–529. 10.1038/nrm2199 17565364

[8] Moore A, Hollien J (2012) The unfolded protein response in secretory cell function. Annu Rev Genet 46: 165–183. 22934644 10.1146/annurev-genet-110711-155644

[9] Li J, Ni M, Lee B, Barron E, Hinton DR, (2008) The unfolded protein response regulator GRP78/BiP is required for endoplasmic reticulum integrity and stress-induced autophagy in mammalian cells. Cell Death Differ 15(9): 1460–1471. 18551133 10.1038/cdd.2008.81 PMC2758056

[10] Pobre KFR, Poet GJ, Hendershot LM (2019) The endoplasmic reticulum chaperone BiP is a master regulator of ER functions: Getting by with a little help from ERdj friends. J Biol Chem 294(6): 2098–2108. 30563838 10.1074/jbc.REV118.002804 PMC6369273

[11] Ryoo D, Stellar H (2007) Unfolding protein response in Drosophilia Why another model can make it fly. Cell Cycle 6(7): 830–835. 17387279 10.4161/cc.6.7.4064

[12] Chou J, Roizman B (1994) Herpes simplex virus 1 γ1 34.5 gene function, which blocks the host response to infection, maps the homologous domain of genes expressed during growth arrest and DNA damage. Proc Natl Acad Sci 91: 5247–5251. 8202476 10.1073/pnas.91.12.5247 PMC43971

[13] Ameri A, Harris L (2008) Activating transcription factor 4. Int J Biochem Cell Biol 40: 14–21. 17466566 10.1016/j.biocel.2007.01.020

[14] Pakos-Zebrucka I, Koryga K, Mnich M, Ljujic A, Samali A, (2016) The integrated stress response. EMBO Rep 17: 1374–1395. 27629041 10.15252/embr.201642195 PMC5048378

[15] Freundt JK, Frommeyer G, Wotzel F, Huge A, Hoffmeier A, (2018) The transcription factor ATF4 promotes expression of cell stress genes and cardiomyocyte death in a cellular model of atrial fibrillation. Biomed Res Int 2018: 1–15. 10.1155/2018/3694362 PMC5996409 30003094

[16] Gwinn DM, Lee AG, Briones-Martin-Del-Campo M, Conn CS, Simpson DR, (2018) Oncogenic KRAS regulates amino acid homeostasis and asparagine biosynthesis via ATF4 and alters sensitivity to L-asparaginase. Cancer Cell 33(1): 91–107. 29316436 10.1016/j.ccell.2017.12.003 PMC5761662

[17] Urano X, Wang A, Bertolotti Y, Zhang P, Chung HP (2000) Coupling of stress in the ER to activation of JNK protein kinases by transmembrane protein kinase IRE1. Science 287: 664–666. 10650002 10.1126/science.287.5453.664

[18] Adams J, Kopp MC, Larburu N, Nowak PR, Ali MMU (2019) Structure and molecular mechanism of ER stress signaling by the unfolded protein response signal activator IRE1. Front Mol Biosci 6:11. 30931312 10.3389/fmolb.2019.00011 PMC6423427

[19] Harding HP, Zhang Y, Bertolotti A, Zeng H, Ron D (2000) Perk is essential for translational regulation and cell survival during the unfolded protein response. Molecular Cell 5: 897–904. 10882126 10.1016/s1097-2765(00)80330-5

[20] Lecca R, Wagner U, Patrignani A, Berger E, Hennet T (2004) Genome-wide analysis of the unfolded protein response in fibroblasts from congenital disorders of glycosylation type-I patients. FASEB J 19(2): 240–2. 15545299 10.1096/fj.04-2397fje

[21] Xu FS, Montgomery MK, Kostas SA, Driver SE, Mello CC (1998) Potent and specific genetic interference by double-stranded RNA in Caenorhabditis elegans. Nature 391: 806–811. 9486653 10.1038/35888

[22] Song M, Rossi JJ (2017) Molecular mechanisms of Dicer: Endoribonuclease an enzymatic activity. Biochem J 474(10): 1603–1618. 28473628 10.1042/BCJ20160759 PMC5415849

[23] Michlewski J, Caceres F (2007) Post-transcriptional control of miRNA biogenesis. RNA 25: 1–16. 10.1261/rna.068692.118 PMC6298569 30333195

[24] Schwarz DS, Tomari Y, Zamore PD (2004) The RNA-induced silencing complex is a Mg2 dependent endonuclease. Curr Biol 14: 787–791. 15120070 10.1016/j.cub.2004.03.008

[25] Mutti S, Louis J, Pappan LK, Pappan K, Begum K, (2008) Protein from the salivary glands of the pea aphid, *Acyrthosiphon pisum*, is essential in feeding on a host plant. Natl Acad Sci 105(29): 9965–9969. 10.1073/pnas.0708958105 PMC2481341 18621720

[26] Avila A, Chandrasekar R, Wilkinson E, Balthazor J, Herman M, (2018) Delivery of lethal dsRNAs in insect diets by branched amphiphilic peptide capsules. J Control Release 273: 139–146. 29407675 10.1016/j.jconrel.2018.01.010 PMC6984438

[27] Miller C, Miyata K, Brown SJ, Tomoyasu Y (2012) Dissecting systematic RNA interference in the red flour beetle Tribolium castaneum: Parameters affecting the efficiency of RNAi. PLoS One 7(10): e47431. 23133513 10.1371/journal.pone.0047431 PMC3484993

[28] Zhang X, Zhang J, Zhu KY (2010) Chitosan/double-stranded RNA particlemediated RNA interference to silence chitin synthase genes through larval feeding in the African malaria mosquito (Anopheles gambiae). Insect Molecular Biology 19: 683–693. 20629775 10.1111/j.1365-2583.2010.01029.x

[29] Whyard S, Singh AD, Wong S (2009) Ingested double-stranded RNAs can act as species specific insecticides. Insect Biochem Mol Biol 39 (2009) 824–832. 19815067 10.1016/j.ibmb.2009.09.007

[30] Akey H, Beck SD (1971) Continuous rearing of the pea aphid, Acrythosiphon pisum on a Holidic diet. Ann Entomol Soc Am 2: 353–356.

[31] Mamedova LK, Robbins L, Johnson BJ, Bradford BJ (2010) Tissue expression of angiopoitin-like protein 4 in cattle. J Anim Sci 88: 124–130. 19783696 10.2527/jas.2009-2258

